# The landscape of knowledge translation interventions in cancer control: What do we know and where to next? A review of systematic reviews

**DOI:** 10.1186/1748-5908-6-130

**Published:** 2011-12-20

**Authors:** Melissa C Brouwers, Kimberly Garcia, Julie Makarski, Lubna Daraz

**Affiliations:** 1 McMaster University, Department of Oncology, Hamilton, Ontario, Canada; 2 McMaster University, Department of Clinical Epidemiology, Hamilton, Ontario, Canada; 3 McMaster University, School of Rehabilitation Science, Hamilton, Ontario, Canada

## Abstract

**Background:**

Effective implementation strategies are needed to optimize advancements in the fields of cancer diagnosis, treatment, survivorship, and end-of-life care. We conducted a review of systematic reviews to better understand the evidentiary base of implementation strategies in cancer control.

**Methods:**

Using three databases, we conducted a search and identified English-language systematic reviews published between 2005 and 2010 that targeted consumer, professional, organizational, regulatory, or financial interventions, tested exclusively or partially in a cancer context (primary focus); generic or non-cancer-specific reviews were also considered. Data were extracted, appraised, and analyzed by members of the research team, and research ideas to advance the field were proposed.

**Results:**

Thirty-four systematic reviews providing 41 summaries of evidence on 19 unique interventions comprised the evidence base. AMSTAR quality ratings ranged between 2 and 10. Team members rated most of the interventions as promising and in need of further research, and 64 research ideas were identified.

**Conclusions:**

While many interventions show promise of effectiveness in the cancer-control context, few reviews were able to conclude definitively in favor of or against a specific intervention. We discuss the complexity of implementation research and offer suggestions to advance the science in this area.

## Background

Innovations in screening and early detection, development of effective treatment interventions, and strategies to improve quality of life have emerged from primary studies, and systematic reviews of these studies, in cancer control [1-9]. These advancements have the capacity to reduce mortality and morbidity from disease. However, optimizing these advancements requires their appropriate application, a goal that is often difficult to achieve [10,11]. Understanding what are the most effective and promising interventions is warranted to ensure that the appropriate options are chosen and incorporated into implementation plans and prioritized for future research studies. The analysis of studies examining the effectiveness of implementation interventions is a key component to an overall knowledge translation (KT) research agenda [12].

The purpose of our study was to conduct a review of systematic reviews to better understand the evidentiary foundation regarding what is known about KT interventions. Specifically, we wanted to better understand the strengths and limitations of the field, to identify what interventions are ready for use now, and to identify research priorities and directions for the future. We were interested in studies conducted in the context of cancer control across the care continuum, from diagnoses to survivorship and end-of-life care, and across cancer diagnoses. We chose this scope given that "context" has been identified as an important consideration in the design and execution of implementation strategies and is a concept central to several KT models and research paradigms [12-15].

## Methods

### Overview

This project, *Knowledge Translation to Improve Cancer Control in Canada*, was funded by the Ontario Institute for Cancer Research and the Canadian Partnership Against Cancer. It received ethics approval from the Hamilton Health Sciences/Faculty of Health Sciences Research Ethics Board, McMaster University, Hamilton, Ontario, Canada. The design, execution, analysis, and reporting of the project was editorially independent from the funders.

To avoid duplication of effort, and to complete the study in the required time, we capitalized on available high-quality databases profiling systematic reviews of interventions to systematically search for, appraise, and evaluate the effectiveness of KT interventions that met our inclusion criteria. Aligning with existing categorical schemes (Canadian Agency for Drugs and Technologies in Health [CADTH] and Cochrane Collaboration's Effective Practice and Organisation of Care [EPOC] group), we sought interventions that targeted the groupings of consumers/patients/public (*i.e.*, consumer interventions), clinicians and healthcare providers (*i.e.*, professional interventions), organizational/managers/system leaders (*i.e.*, organizational interventions), regulatory (*i.e.*, new health service delivery regulation), and financial (*i.e.*, incentives).

### Search

To identify existing systematic reviews that met our inclusion criteria, we used three databases as our sources:

1. Rx for Change (CADTH-EPOC Group Collaborative Initiative): http://www.cadth.ca/en/resources/rx-for-change/database

2. Health Systems Evidence (formerly, Program in Policy Decision-Making), McMaster University: http://www.healthsystemsevidence.org/

3. McMaster KT+, McMaster University: http://plus.mcmaster.ca/kt/Default.aspx

### Eligibility criteria

#### Inclusion criteria

• Study designs: Systematic reviews published between 2005 and 2010. While the methodology is still evolving, this interval was chosen to align with best evidence suggesting median survival time of a systematic review is approximately five years [16].

• Outcomes: Systematic reviews that included at least one of the following outcomes: measurable clinical outcomes, observable behavior change, documented intention to behave, documented attitudes, documented knowledge uptake, or reported stakeholder satisfaction.

• KT interventions: Interventions that focused on at least one of the following target groupings: consumer, professional, organization, regulatory, and financial, as outlined in the CADTH-EPOC framework.

• Clinical scenarios:

○ Diagnosis: Interventions that were tested in cancer-specific environments (any cancer diagnosis). Reviews that included cancer as part of the clinical context or that were generic/nonspecific to clinical condition were also considered.

○ Stage of continuum of care: Diagnosis, treatment, follow-up, survivorship, end-of-life [17]^a^.

#### Exclusion criteria

• Study designs: Any design other than systematic reviews.

• Language: Non-English language due to cost and availability of translations.

### Data extraction

Data were extracted from the systematic reviews meeting our eligibility criteria. For all reviews, data extracted included (i) intervention name/label; (ii) intervention definition and purpose; (iii) theories, frameworks, or models used to inform the design or execution of the intervention; (iv) key operational elements underpinning the execution of the intervention; (v) setting; (vi) geography (where the intervention was tested); (vii) stakeholder involvement (who and type of involvement); (viii) evaluation strategy; (ix) measurement instrument(s); (x) quality indicators and outcomes; (xi) recommendations and conclusions; (xii) cost information and/or information regarding sustainability of the intervention; and (xiii) AMSTAR quality appraisal (if available).

### Quality control and critical appraisal

Title, abstract, and full-text screenings were done in duplicate (KG and LD). Disagreements were resolved by consensus or by a third party (JM). Data extraction and critical appraisal (if required) were conducted by one researcher and audited by a second. In circumstances where appraisal was not completed by the original source, the systematic reviews were appraised using AMSTAR [18], an 11-item evaluation tool assessing methodological standards, presentation, and critical appraisal in systematic reviews. AMSTAR ratings can range between 1 and 11, with 11 denoting highest quality.

### Interpretation of systematic reviews' results and research priorities/direction

Data tables describing the systematic review study characteristics and results were circulated to subgroups of the research team (see Additional File 1). For each assigned systematic review and using a 7-point scale, members of the subgroups were asked to indicate their "assessment of effectiveness" for each intervention reviewed based on their interpretation of the data. A rubric was designed to guide these assessments and the interpretation of the rating scale. Interventions rated between 1 and 3 were considered ineffective, interventions rated 4 and 5 were considered promising and worth additional investigation, and interventions rated 6 or 7 were considered effective. Average scores of ratings were calculated for each systematic review. Investigators were then asked to identify any specific research priorities or research questions they thought relevant.

## Results

### Overall

The initial search for systematic reviews yielded 591 reviews for consideration from the three source databases. Of these, a total of 38 reviews were originally considered and 31 retained [19]^b^. An additional three reviews were included post hoc, identified by members of the research team (see Additional File 2).

In total, 34 unique systematic review publications providing 41 evidence summaries on 19 unique interventions comprised the evidence base. Table 1 provides the definitions of the interventions considered. Some systematic reviews addressed more than one intervention or provided more than one evidence summary; in addition, more than one systematic review was available for some of the interventions. Additional File 2 provides the references for the included systematic reviews.

**Table 1:** Intervention definitions

Intervention	Definition
*Consumer interventions*	

Education	interventions informing patients about their treatment and their health (education, information provision, promotion of health)

Decision aids/shared decision-making interventions	interventions designed to assist patients make specific and deliberative choices among options by providing information on the options and outcomes relevant to a person's health status

Interactive health communication application (IHCA) interventions	interventions aimed at enabling interactions between an individual and a communication technology to access or transmit health information, to receive guidance, or to receive support

Contracts	a behavioral strategy aimed at improving patient adherence by setting out a set of rules regarding the behavior of interest and formalizing commitment to adhere to the rules

Reminder packaging	interventions aimed to facilitate safe and appropriate medication use (*e.g.*, monitored dose symptoms, multi-compartment aids)

Multifaceted interventions	two or more interventions aimed at assisting patients with adherence to treatment/medications and improving the prescription process

*Professional interventions*	

Educational outreach and audit and feedback	interventions whereby a trained person meets with providers in their practice setting to give information with the goal to change clinical behavior (also referred to as academic detailing) OR any summary of clinical performance (from health records, observation, computer systems) of healthcare over a specified time that may also include recommendations for clinical action

Clinical decision support systems (IT/IM/informatics)	information system interventions that provide the clinician with decision support, including critical clinical data, reminders, advice on drug or care provision, etc.

Computerized physician order entry (IT/IM/informatics)	computer-based systems for ordering medications with automated aspects to the ordering process, such as a list of possible medications for a physician to choose, drug interaction or contraindication prompts, reminders, etc.

Tracker/reminder systems (IT/IM/informatics)	specific interventions that prompt healthcare providers with information specific to the patient or the encounter that would advise on action to do or action to avoid (interventions can be verbal, paper, or electronic)

Local opinion leaders	educational leaders and influentials nominated by their colleagues

Tailored interventions	identify barriers to change and subsequent design of an intervention that addresses identified barriers

Clinical pathways	document-based tools that provide a link between best available evidence and clinical practice by providing recommendations, processes, and time-frames for the management of specific medical conditions or interventions

Guidelines for professions allied to medicine	a systematic statement aimed at assisting in decisions by providers and patients for a specific clinical condition

Discharge planning from hospital to home	interventions aimed at providing individualized plans as a patient is moved from hospital to home

*Organizational interventions*	

Changing length of consultation	interventions designed to increase consult time between primary care provider and patient

Routine standard assessment interventions	interventions designed to improve the assessment and documentation of patients (akin to pathology checklist concept in Ontario or surgical checklist concept in various provinces)

Chronic care model interventions	interventions aimed at redesigning ambulatory care by modifying elements of the chronic care model (elements include self-management support, decision support, delivery system design, clinical information systems, healthcare organizations, and community resources)

Shared-care interventions	any type of structured system that involves continuing collaborative clinical care between primary care and specialty care in the management of patients

Shared-care tactic interventions	tactics aimed to facilitate information sharing between providers who provide care to a patient; include (i) liaison meetings--meetings between specialists and primary care teams whereby ongoing management of patients within the service is planned and discussed, (ii) shared-care record cards--a formal information-sharing arrangement where a set of data is agreed to, entered onto a record card, and usually carried by the patient, or (iii) computer-assisted shared care/email--a formal information-sharing arrangement whereby a data set is agreed to, entered onto a record card, and shared between two sectors on computer (can also include coordinated computer registration and patient recall)

Health information technology interventions	switching the format or structure of the medical record, such as computerized medical records

IT = information technology; IM = information management.

The overall quality of the systematic reviews, as measured by the AMSTAR tool [18], was moderate. The systematic reviews were most apt to target the treatment stage of the continuum and least likely to target survivorship (Table 2). Further, despite being a highly prevalent disease site, few systematic reviews targeted patients with lung cancer (Table 3). Some less prevalent diagnoses (head and neck, sarcoma, and melanoma) were rarely or never targeted. A significant number of reviews did not specify cancer diagnosis.

**Table 2:** Systematic reviews: Stage in continuum of cancer care and implementation intervention cluster

Cluster	Continuum-of-care stage							
	**Prevention**	**Screening**	**Diagnosis**	**Treatment**	**Survivorship**	**Follow-up**	**Supportive care**	**Palliative end-of-life care**

Professional	5	7	7	10	1	2	2	2

Consumer	4	2	8	16	1	3	8	4

Organizational	1	3	6	8	1	3	4	3

Financial	0	0	0	0	0	0	0	0

Regulatory	0	0	0	0	0	0	0	0

**Table 3:** Systematic reviews: Cancer diagnosis and implementation intervention cluster

				Cluster		
		
		Professional	Consumer	Organizational	Financial	Regulatory
	Breast	4	9	4	0	0
	
	Gastrointestinal	2	2	2	0	0
	
	Genitourinary	2	4	2	0	0
	
	Gynecological	1	1	1	0	0
	
	Head and neck	0	2	0	0	0
	
**Diagnosis**	Hematologic	1	2	1	0	0
	
	Lung	0	3	0	0	0
	
	Melanoma	1	1	1	0	0
	
	Neuro-oncology	0	0	0	0	0
	
	Sarcoma	1	0	1	0	0
	
	Not specified	5	6	5	0	0

With respect to characteristics of the systematic reviews (Table 4), most did not specify whether a KT theory, model or framework was used to inform the design of the project or the intervention itself. Most of the reviews used a mixture of study designs as their primary evidentiary source, and only four reviews used non-randomized controlled trial (RCT) data exclusively as their primary evidence. Most systematic review authors reported poorly executed and poorly reported primary studies.

**Table 4:** Characteristics of systematic reviews

Type of intervention (number of evidence summaries)	Theory/framework			Study designs included			Outcomes included					
	
	Y	N	NS	RCT only	Non-RCT only	Mixed	C	BO	BI	K	A	S
*Consumer interventions*												

Patient education/patient information (n = 8)	2	0	6	2	0	6	4	3	6	6	4	5

Decision aids/shared decision making (n = 5)	2	2	1	0	0	5	1	3	2	4	1	3

Interactive health communication applications (n = 1)	0	0	1	1	0	0	1	1	0	1	0	0

Contracts (n = 1)	1	0	0	1	0	0	0	1	0	1	0	1

Reminder packaging (n = 1)	0	0	1	1	0	0	0	1	0	0	1	0

Multifaceted (n = 1)	0	0	1	1	0	0	1	1	0	0	0	0

***Overall***	***5***	***2***	***10***	***6***	***0***	***11***	***7***	***10***	***8***	***12***	***6***	***9***

*Professional interventions*												

Educational outreach visits & audit and feedback (n = 4)	1	2	1	2	1	1	4	2	0	2	0	1

IT/IM/informatic interventions (n = 5)	0	1	4	0	1	4	4	5	0	0	0	0

Local opinion leaders (n = 1)	0	0	1	1	0	0	0	1	0	0	0	0

Tailored interventions (n = 1)	1	0	0	1	0	0	1	0	0	0	0	0

Clinical pathway interventions (n = 1)	--	--	--	0	0	1	1	1	0	0	0	0

Guidelines for professions allied to medicine (n = 1)	--	--	--	0	0	1	0	1	0	0	0	0

Discharge planning from hospital to home (n = 1)	--	--	--	1	0	0	1	0	0	0	0	1

***Overall***	***2***	***3***	***6***	***5***	***2***	***7***	***11***	***10***	***0***	***2***	***0***	***2***

*Organizational interventions*												

Changing length of consultation (n = 1)	0	0	1	0	0	1	1	1	0	0	0	1

Routine standardized assessment (n = 1)	0	0	1	0	0	1	1	1	0	1	1	1

Chronic care model interventions (n = 1)	1	0	0	0	0	1	0	1	0	0	0	0

Models-of-care/integrated care-related interventions (n = 5)	0	2	3	0	2	3	4	2	1	3	1	3

Shared-care tactic interventions (n = 1)	0	1	0	0	0	1	1	1	0	0	0	1

Health information technology (n = 1)	1	0	0	0	0	1	1	1	0	0	0	1

***Overall***	***2***	***3***	***5***	***0***	***2***	***8***	***8***	***7***	***1***	***4***	***2***	***7***

Y = yes; N = no; NS = not specified; RCT = randomized controlled trial; C = clinical; BO = behaviour observed; BI = behaviour intention; K = knowledge; A = attitudes; S = satisfaction; IT = information technology; IM = information management.

Note: The reviews added post-hoc (Rotter, 2010; Shepperd, 2010; Thomas, 2009) did not have full data extraction completed; this is indicated by cells with "--".

Common outcomes reported in the literature were measures of knowledge, satisfaction, and observed behavior and, to a lesser extent, clinical outcomes. Indeed, 7 of 17 consumer evidence summaries reported clinical outcomes, while 12 reported on knowledge, 10 reported on behavior observed, 9 reported on satisfaction, 8 reported on behavior intention, and 6 reported on attitude. For professional evidence summaries, 11 of 14 reported clinical outcomes, compared to knowledge (2), satisfaction (2), and behavior observed (10). Similarly, the most reported outcome for organizational evidence summaries were clinical outcomes (8), compared to behavior observed (7), satisfaction (7), knowledge (4), attitude (2), and behavior intention (1). However, when considering across the 41 evidence summaries in our sample, only 15 of 41 reported clinical outcomes.

With respect to intervention context (Table 5), clinicians were most often delivering the intervention, and a clinical environment (*e.g.*, clinics, hospitals) was the most common setting for the intervention to take place. Interventions were most often delivered in person, by paper, or by phone. Technology-based modalities were used less often unless they defined the intervention itself (*e.g.*, electronic medical record, clinical decision support systems).

**Table 5:** Characteristics of interventions studied

Type of intervention (number of evidence summaries)	Context: Who delivered						Context: Where delivered				Context: How delivered					
	
	Pt	Fam	Clin	Ad	PH	O	Home	Comm	ClE	O	Per	Paper	Phone	TI	TS	NS
*Consumer interventions*																

Patient education/patient information (n = 8)	0	0	5	0	0	3	4	3	7	4	4	7	4	2	2	0

Decision aids/shared decision making (n = 5)	0	0	5	0	0	0	2	0	3	2	2	5	1	4	2	0

Interactive health communication applications (n = 1)	0	0	0	0	0	1	1	1	1	0	1	1	0	1	1	0

Contracts (n = 1)	0	0	1	0	0	0	1	0	1	0	1	1	0	0	0	0

Reminder packaging (n = 1)	1	1	1	0	0	1	1	1	1	1	1	0	0	0	1	0

Multifaceted (n = 1)	1	1	1	0	0	1	1	1	1	1	1	1	1	1	1	0

***Overall***	***2***	***2***	***13***	***0***	***0***	***6***	***10***	***6***	***14***	***8***	***10***	***15***	***6***	***8***	***7***	***0***

*Professional interventions*																

Educational outreach visits & audit and feedback (n = 4)	0	0	3	1	0	2	0	1	4	1	4	4	3	2	1	0

IT/IM/informatic interventions (n = 5)	0	0	2	1	0	4	0	2	5	1	0	0	0	4	1	0

Local opinion leaders (n = 1)	0	0	1	0	0	0	0	0	1	0	1	0	0	0	0	0

Tailored interventions (n = 1)	0	0	0	0	0	1	0	1	1	0	0	0	0	0	0	1

Clinical pathway interventions (n = 1)	0	0	1	0	0	0	--	--	--	--	--	--	--	--	--	--

Guidelines for professions allied to medicine (n = 1)	0	0	1	0	0	0	--	--	--	--	--	--	--	--	--	--

Discharge planning from hospital to home (n = 1)	0	0	1	0	0	0	--	--	--	--	--	--	--	--	--	--

***Overall***	***0***	***0***	***9***	***2***	***0***	***7***	***0***	***4***	***11***	***2***	***5***	***4***	***3***	***6***	***2***	***1***

*Organizational interventions*																

Changing length of consultation (n = 1)	0	0	1	0	0	0	0	0	1	0	1	0	0	0	0	0

Routine standardized assessment (n = 1)	0	0	1	0	0	1	0	0	1	0	1	1	0	0	0	0

Chronic care model interventions (n = 1)	0	0	1	0	0	1	0	1	1	0	1	0	0	0	0	1

Models-of-care/integrated care-related interventions (n = 5)	0	0	5	0	0	0	1	2	5	1	5	4	2	2	0	0

Shared-care tactic interventions (n = 1)	0	0	1	0	0	0	1	1	1	1	1	1	1	1	0	0

Health information technology (n = 1)	0	0	1	1	0	0	0	0	1	0	0	0	0	1	0	0

***Overall***	***0***	***0***	***10***	***1***	***0***	***2***	***2***	***4***	***10***	***2***	***9***	***6***	***3***	***4***	***0***	***1***

Pt = patients; Fam = family; Clin = clinicians; Ad = administrator/manager; PH = public health; O = other; Comm = community; ClE = clinical environment; Per = person; TI = technology--interactive; TS = technology--static; NS = not specified; IT = information technology; IM = information management.

Note: The reviews added post-hoc (Rotter, 2010; Shepperd, 2010; Thomas, 2009) did not have full data extraction completed; this is indicated by cells with "--".

The following sections, including the tables and additional files, provide an overview of the results by cluster of main intervention groupings. For more information on the complete raw data set, readers are asked to contact the corresponding author.

### Interventions aimed at consumers (Additional File 3)

Sixteen publications addressing six unique consumer interventions provided 17 evidence summaries. Consumer interventions included education/information provision, decision-making aids, and interventions to support behavior change. Tables 4, 5, and 6 summarize characteristics of the systematic reviews; Table 7 summarizes the quality and efficacy appraisals of the interventions; and Table 8 summarizes the research suggestions made by members of the team.

**Table 6:** Consumer-focused interventions: Number of systematic reviews for each cluster

Type of intervention	Total number of SRs	Number of SRs--cancer only	Number of SRs-- mixed	Number of SRs-- no cancer
**Patient education and patient information**	8	4	4	0

**Patient decision aids**	5	3	2	0

**Interactive health communication applications**	1	0	1	0

**Contracts**	1	0	0	1

**Reminder packaging**	1	0	0	1

**Multifaceted**	1	0	1	0

SR = systematic review.

**Table 7:** Consumer interventions: Appraisal of systematic review (AMSTAR) scores and intervention effectiveness ratings by research team

Intervention cluster and systematic review first author (year)	Scores and ratings		
	
	AMSTAR (1 to 11)	Research team ratings	
		Mean	SD
**Patient education/patient information**			
Bennett (2009) [26]	8	5.7	0.67
Gaston (2005a) [35]	5	4.7	0.45
Gysels (2007) [37]	5	3.0	1.00
Goldberg (2007a) [36]	3	3.8	0.45
Wofford (2005) [56]	7	4.3	0.84
Santo (2005) [47]	7	4.6	0.89
Conn (2008) [30]	10	4.3	1.79
Raynor (2007) [45]	8	4.0	0.71
Overall mean score	7	4.3	

**Patient decision aids**			
Evans (2005) [33]	7	4.4	1.14
Gaston (2005b) [35]	5	4.3	0.97
Waljee (2007) [54]	8	6.1	0.22
Edwards (2008) [32]	5	3.8	0.45
Joosten (2008) [41]	5	5.1	0.22
Overall mean score	6	4.7	

**Interactive health communication applications**			
Murray (2005) [43]	11	6	1

**Contracts**			
Bosch-Capblanch (2007) [27]	9	3.8	0.84

**Reminder packaging**			
Heneghan (2006) [39]	10	5.1	0.74

**Multifaceted**			
Haynes (2008) [38]	10	4.6	0.89

**Table 8:** Consumer interventions: Nominated research ideas

Type of intervention	Research ideas
Patient education/patient information	Randomized studies directly comparing different intervention formats/modalities/techniques on satisfaction, adherence, and clinical outcomes (prioritize those interventions from which individual studies have shown greatest promise on clinical outcomes) (*i.e.*, how to do it)

Decision aids	Development, testing, and evaluation of cancer decision aids in understudied areas (*e.g.*, role within personalized medicine, molecular-genetics) •
	Development, testing, and evaluation of cancer decision aids in understudied populations

Interactive health communication applications (IHCA)	What are the implications for IHCA on privacy policies and legislations?

Contract interventions	Outside of cancer prevention, no role

Reminder packaging	Research to identify if adherence to medication protocols is a problem for cancer patients (*e.g.*, for which drugs, cancer diagnoses, complexity of drug regimen) •
	Generalize to other aspects of cancer care--What are effective strategies to remind cancer survivors of follow-up and monitoring regimens? (linked to discharge plan priorities)

Multifaceted intervention	Design and evaluate multifaceted consumer interventions for specific cancer control problems using Haynes *et al. *review as foundation (*i.e.*, deconstruct systematic review to identify most promising intervention clusters for specific contexts)

Systematic review priorities	Acquiring skills and competencies •
	Consumer system participation •
	Minimizing risks or harms

The overall quality of the systematic reviews targeting consumer interventions was variable, ranging from poor to high. The average AMSTAR score was 7, with scores ranging from 3 to 10. Most of the systematic reviews did not include a meta-analysis or an empirical synthesis of findings. Evidence of effectiveness was most promising for patient education (*e.g.*, improvements in patient knowledge and clinical outcomes); decision aids (*e.g.*, improvements in patient knowledge and satisfaction, reduction in patient decisional conflict, and impact on decisions); and interactive health communication applications (*e.g.*, improvements in knowledge, social support, behavior, and clinical outcomes). Most reviews within a given intervention type yielded significant variability with respect to effectiveness, and most authors were unable to provide definitive conclusions to their use.

Assessments by research team members on effectiveness yielded overall mean scores (across evidence summaries of similar interventions) ranging between 3.8 and 6.0 (see "Team Ratings" column of Table 7). Only one, interactive health communication applications, was rated as being effective. The remaining interventions were rated as promising and candidates for more study. Eight research ideas and three systematic review topics were identified by members of the research team (Table 8).

### Interventions aimed at professionals (Additional File 4)

Twelve publications addressing seven unique professional interventions provided 14 evidence summaries. Professional interventions included education, audit and feedback, information technology (IT)/information management (IM)/informatics, clinical decision support systems, computerized physician order entry, reminders, local opinion leaders, tailored interventions, clinical pathways, guidelines, and discharge planning. Tables 4, 5, and 9 summarize characteristics of the systematic reviews; Table 10 summarizes the quality and efficacy appraisals for the interventions; and Table 11 summarizes the research suggestions made by members of the research team.

**Table 9:** Professional-focused interventions: Number of systematic reviews for each cluster

Type of intervention	Total number of SRs	Number of SRs--cancer only	Number of SRs-- mixed	Number of SRs-- no cancer
**Educational outreach visits & audit and feedback**	4	2	2	0

**IT/IM/informatics**	5	1	4	0

**Local opinion leaders**	1	0	1	0

**Tailored interventions**	1	0	0	1

**Clinical pathways**	1	0	1	0

**Guidelines for professions allied to medicine**	1	0	1	0

**Discharge planning from hospital to home**	1	0	1	0

SR = systematic review; IT = information technology; IM = information management.

**Table 10:** Professional interventions: Appraisal of systematic review (AMSTAR) scores and intervention effectiveness ratings by research team

Intervention cluster and systematic review first author (year)	Scores and ratings		
	
	AMSTAR (1 to 11)	Research team ratings	
		Mean	SD
**Educational outreach visit & audit and feedback interventions**			
Goldberg (2007b) [36]	3	4.6	0.89
O'Brien (2007) [44]	8	4.8	1.17
Goldberg (2007c) [36]	3	3.8	0.98
Jamtvedt (2006) [40]	8	4.7	0.82
Overall mean score	5.5	4.5	

**IT/IM/informatic interventions**			
Goldberg (2007d) [36]	3	3.7	0.52
Garg (2005) [34]	5	5.3	0.52
Ammenwerth (2008) [23]	6	5.7	0.52
Beach (2006a) [25]	5	5.7	1.03
Shojania (2009) [50]	8	4.7	0.52
Overall mean score	5.4	5.0	

**Local opinion leader interventions**			
Doumit (2007) [31]	7	4.3	0.82

**Tailored interventions**			
Baker (2010) [24]	7	4.8	1.47

**Clinical pathway interventions**			
Rotter (2010)^a ^[46]	--	NA	NA

**Guidelines for professions allied to medicine**			
Thomas (2009)^a ^[53]	--	NA	NA

**Discharge planning from hospital to home**			
Shepperd (2010)^a ^[49]	--	NA	NA

^a^These reviews were included *post hoc*, identified by members of the research team.

IT = information technology; IM = information management.

**Table 11:** Professional interventions: Nominated research ideas

Type of intervention	Research ideas
Educational outreach visits (EOV)/audit and feedback (AF)	Better quality trials directly evaluating specific modalities and methods of EOV (proper randomization, baseline data, variety of outcomes--including patient outcomes and costing)
	Better quality trials directly evaluating specific modalities and methods of methods of AF (as above)
	For which clinicians, clinical conditions, and stage in the continuum is EOV most impactful?
	For which clinicians, clinical conditions, and stage in the continuum is AF most impactful?
	What is the impact of regional AF versus individual AF on changing patterns of practice?

IT/IM/informatic interventions	Better quality trials directly evaluating specific modalities and methods of informatic interventions (proper randomization
	For which clinicians, clinical conditions, and stage in the continuum are informatics interventions most impactful?
	Develop methods to enable practice guidelines to be directly integrated into informatic interventions
	What is the cost effectiveness of different informatic interventions?
	Are informatic interventions effective in non-drug-prescribing aspects of cancer care?

Local opinion leader interventions	Methodological development in choosing local opinion leaders in a reliable manner
	Methodological development in understanding factors that increase and decrease sustainability of local opinion leader designation
	Research to better understand for which provider groups and under what clinical contexts (cancer diagnosis, practice setting, stage of the cancer trajectory) local opinion leaders are most effective

Tailored interventions	Methodological analysis of the operational techniques of tailoring in existing high-quality primary studies
	Methodological development to determine when tailoring has or has not addressed identified barriers
	Identification of defining factors of effective tailoring
	Direct comparisons of different tailoring interventions (tactic and modality, etc.) on uptake of evidence, processes of care, and clinical outcomes

Clinical pathway interventions	Testing of clinical pathway interventions on different stages of continuum of cancer care
	Testing of clinical pathway interventions with different healthcare providers involved in cancer control
	Methodological development to determine for which clinical problem (*e.g.*, cancer diagnosis, complexity of care) clinical pathways are most effective
	Direct comparisons of different clinical pathway strategies (methods and modality, etc.) on uptake of evidence, processes of care, and clinical outcomes
	Is the introduction of clinical pathways cost effective in Ontario/Canada?

Guidelines	Compare and contrast use of and impact of guidelines on processes of care and clinical outcome as a function of cancer care provider
	Testing innovative strategies to disseminate guideline messages to different providers
	Testing of innovative strategies to disseminate guideline messages to administrators
	Testing of innovate strategies to disseminate guideline messages to policy makers

Discharge planning	What are the defining features/components to a discharge plan that are linked to patient satisfaction, provider satisfaction, process outcomes, and clinical outcomes?
	Determining the specific clinical components for discharge plans for different cancer diagnoses

IT = information technology; IM = information management.

For these systematic reviews, the average AMSTAR score was 6, with scores ranging from 3 to 8. Trials that comprised the reviews included RCTs, clinical controlled trials, pre-/post studies, cluster RCTs, time series, observational, and trials labeled as "other." Of the 14 evidence summaries, 4 undertook quantitative pooling (*i.e.*, meta-analysis).

Evidence of effectiveness was most promising for educational outreach and audit and feedback interventions (median improvement in clinical outcomes 5%); clinical decision support (improved clinical performance); computer order entry (reduction in medical errors); clinical pathways (reduction in complication rates); local opinion leaders (reduction in clinician noncompliance); and tailored interventions (improvement in some clinical outcomes). However, these benefits are contrasted against the concerns with the overall quality and lack of consistency across the systematic reviews. Moreover, the primary studies included in the systematic reviews are reported to be of poor quality, heterogeneous, and poorly reported with respect to the interventions, contexts, and measurements of outcomes. Together, this makes definitive conclusions about professional interventions very challenging.

Research team ratings of the interventions were assessed, and the overall mean scores (across evidence summaries of the same intervention) ranged between 4.3 and 5.0 all were within the "promising" category (see "Team Ratings" column in Table 10). Twenty-eight research ideas were recommended by members of the research team (see Table 11).

### Interventions aimed at organizations (Additional File 5)

Nine reports addressing six unique organizational interventions provided 10 evidence summaries. The interventions included organizational/structural specific, continuity-of-care related, shared-care tactics, revisions of professional roles, and health information technology. Tables 4, 5, and 12 summarize characteristics of the systematic reviews; Table 13 summarizes the quality and efficacy appraisals of the interventions; and Table 14 summarizes the research suggestions made by members of the team.

**Table 12:** Organizational-focused interventions: Number of systematic reviews for each cluster

Type of intervention	Total number of SRs	Number of SRs--cancer only	Number of SRs-- cancer included	Number of SRs-- no cancer
**Changing length of consultation**	1	0	0	1

**Routine standard assessment**	1	1	0	0

**Chronic care model**	1	0	1	0

**Models of care/integrated care**	5	2	3	0

**Shared-care tactic**	1	0	1	0

**Health information technology**	1	0	1	0

SR = systematic review.

**Table 13:** Organizational-focused interventions: Appraisal of systematic review (AMSTAR) scores and intervention effectiveness ratings by research team

Intervention cluster and relevant systematic review first author (year)	Scores and ratings		
	
	AMSTAR (1 to 11)	Research team ratings	
		
		Mean	SD
**Changing length of consultation interventions**			
Wilson (2006) [55]	7	3	0.71

**Routine standard assessment interventions**			
Goldberg (2007e) [36]	3	3.8	1.30

**Chronic care model interventions**			
Coleman (2009) [29]	2	4.4	0.55

**Models-of-care/integrated care-related interventions**			
Lewis (2009) [42]	7	3.8	1.10
Smith (2008) [52]	8	3.2	1.30
Beach (2006b) [25]	5	4.6	0.89
Goldberg (2007f) [36]	3	3.6	0.89
Scheuner (2008) [48]	4	2.6	0.55
Overall mean score	5.4	3.6	

**Shared-care tactic interventions**			
Smith (2007) [51]	8	3	1.22

**Health information technology interventions**			
Chaudhry (2006) [28]	4	4.6	0.89

**Table 14:** Organizational interventions: Nominated research ideas

Type of intervention	Research ideas
Changing length of consultation	Does length of consultation influence patient satisfaction, clinician satisfaction, or patient outcomes in a clinical context?
	Modeling the clinical encounter to predict patient satisfaction, clinician satisfaction, and patient outcomes. Use length of consult time as one of the predictors (examples of others: types of information shared, type of clinician, diagnosis).
	In what stages of the cancer continuum does the length of consultation impact patient satisfaction, clinician satisfaction, and patient outcomes?
	What is the cost effectiveness of longer consultation times?

Routine assessment interventions	Conduct a high-quality systematic review examining the impact of routine standard assessment (across cancer care continuum) on delivery of cancer care, satisfaction (patient and clinician), and clinical outcomes
	What is the impact of routine standard assessment on other aspects of cancer care other than pain?
	Conduct a well-designed randomized trial to evaluate the impact of routine standard assessments on delivery of care (fidelity), satisfaction of care (patient and clinician), and clinical outcomes
	Compare and contrast methods used to determine and create tools to support implementation of routine standard assessments to ensure they are based on evidence and acceptable to clinicians and patients
	Research examining how to implement routine standard assessments into an oncology practice setting (ambulatory or in-patient) so that it is acceptable (to management, clinicians, patients), effective, and cost effective

Chronic care model (CMM) interventions	Conduct a high-quality systematic review examining the impact of CMM on delivery of cancer control, satisfaction (patient, clinician, policy), and clinical outcomes
	Research (qualitative and/or scoping review) to assess whether CCM applies well to the cancer control context. Which components of the CCM (if any) are most relevant to cancer control? Which aspect of cancer control (*e.g.*, diagnosis) lends itself to the CCM model?
	Design, evaluate, and refine tools to support each of the six CCM components using high-quality methods

Models of care/integrated care interventions 1	Research to better understand for what cancer diagnoses, cancer care options, stages in the continuum, and contexts (*e.g.*, geography) are different models of care and integration of services most appropriate (*e.g.*, systematic review, case study intervention)
	High-quality economic analysis comparing different models of care
	Research aimed to analyze (and perhaps statistically model) existing models of care to better understand the mechanisms underlying the processes and the outcomes of different approaches
	Deconstruction of existing systematic reviews to better understand the mechanisms underlying different models of care
	Develop methods to better measure the concepts of shared care and integrated care

Models of care/integrated care interventions 2	Development/identification, implementation, and evaluation (process, satisfaction, patient outcomes) of various models (care and service) aimed at the diagnostic stage of continuum and transition to treatment (*e.g.*, systemic review, scoping review, or randomized trial)
	As above but focused on treatment only (*e.g.*, systematic review or scoping review)
	As above but aimed at the treatment stage of the continuum and transition to survivorship or palliative care
	How to best introduce new models of care or new clinical roles into the care system
	Research aimed to test effectiveness, safety, satisfaction (patient, providers, and system) and cost effectiveness of new clinical roles in cancer control by non-medical clinical professionals

Shared-care implementation tactic interventions	Using high-quality randomized methods, compare and contrast different existing tactics aimed to facilitate communication between healthcare providers
	Develop, test, and refine new tactics aimed at facilitating communication between different practitioners

Health information technology (HIT) interventions	What are the most effective and efficient strategies to implement an HIT solution?
	For what cancer-control contexts (*e.g.*, continuum of care, diagnosis, practice setting) is an HIT solution most appropriate?
	Does embedding evidence-based recommendations into the HIT solutions improve quality of care over HIT solutions alone?
	Are HIT solutions cost effective?

Organizational interventions are those aimed at encouraging use and uptake of knowledge at the organizational level. The average AMSTAR score was 5, with scores ranging from 2 to 8, which indicates the range of very poor methodological quality to moderately high methodological quality. Overall, the reviews were not able to provide definite conclusions (*e.g.*, statistically significant findings) to support the use of any of the specific interventions reviewed.

Two of the interventions, changing length of consultation time and shared-care tactic interventions, were rated by members of the research team as not effective (ratings of 3 and under). The remaining interventions fell between 4 and 5 on the scale, indicating promise and are candidates for further study (see Table 13). Twenty-eight research ideas were recommended by members of the research team (see Table 14).

## Conclusion

We considered 34 unique systematic reviews providing 41 evidence summaries for 19 KT interventions. The quality of the execution of the systematic reviews varied significantly, with AMSTAR ratings ranging between 2 and 10. Primary evidence serving as the foundation for the systematic reviews included RCTs and nonrandomized trials. Systematic review authors describe the primary evidence as uneven with respect to quality, reporting, and outcomes.

While many interventions suggested promise of effectiveness, few reviews were able to conclude definitively in favor of or against a specific intervention. The interpretation of the evidence by members of the research team aligned with this analysis. The majority of the interventions were rated by the research team members as promising, but in need of additional study.

In considering KT in cancer control, one is struck by the complexity of the enterprise. While there are many studies being conducted, the quality is decidedly uneven and the impact on patient care or system performance is questionable. To that end, some key observations are noted and conclusions for the research enterprise can be drawn.

Overall, the approach to KT in cancer control appears patchy and unsystematic. While this may be due, in part, to the breadth, size, and scope of the research area, it is likely to be a major contributor to the hodgepodge of studies being conducted, the failure of the research community to consistently embrace high-quality research paradigms and standards, and the inability to create a common language and taxonomy in the field. Indeed, common across the systematic reviews considered here is that the studies that underpin them often fail to adequately describe all aspects of the KT intervention under investigation and (where relevant) the control group. This makes it very difficult to synthesize data, to improve the overall research enterprise, and to build from one study to another. Here, work by Cochrane's EPOC group and researchers such as Michie and others may assist in designing a common language, a common set of operational definitions, and common labels to facilitate the advancement of the KT field [20-22].

The complexity of the cancer field and the impact of that complexity to the KT research agenda cannot be underestimated. The numerous but unique diagnoses, the variety of providers involved in cancer care and control (*i.e.*, public health, primary care, medical specialists, allied health providers, lay and peer providers), the various organizational settings in which care is offered, the risks associated with some care options, and the variability in decision-making styles by individuals affected by cancer are examples of this complexity. As it relates to the KT research enterprise, the role of context and individual differences must be stressed.

The design and execution of the primary studies fall below acceptable levels of quality. For example, we found that the systematic reviews often fail to measure meaningful end points because these data are not available in the primary literature that comprise the evidentiary base for synthesis. While measures of knowledge, satisfaction, and intention are important--and in fact, better studied--measures of intervention fidelity (or adherence to intervention), relevant clinical end points, and valid patient-centered outcomes are often lacking.

In addition, the design and execution of the systematic reviews in the KT field are uneven. For example, use of factorial designs, multilevel modeling techniques, and regression strategies could improve the precision by which we understand KT interventions. The application of these techniques is warranted. While this has been undertaken in some of the systematic reviews, it is not consistent, nor is it being done in the primary studies underpinning the existing reviews.

Thus, common principles of good scholarship and methodological rigor are required in systematic reviews of KT research and in the primary studies that underpin them. Namely, well-defined research questions, appropriate research design, patient-centered outcomes, analytic strategies to better understand the mechanisms associated with change, and completeness of reporting are necessary.

There are, however, limitations to our study. First, to manage scope and resource constraints of the project, we considered only systematic reviews available in databases of systematic reviews. While trying to avoid duplication, we acknowledge there may be other reviews that would have met our inclusion criteria that were not included in any of the three databases. Further, one review by Grimshaw (2006) [19] was found to be eligible during our search but was erroneously deleted and not included in our review. In addition, we acknowledge that there are likely primary studies not yet included in any of the systematic reviews we considered that would have been relevant to our question.

Second, this study was Canadian in focus with respect to members of the research team. While this conformed to the *request for proposals *criteria of the project's funders, it may be that a different composition of individuals would have yielded different conclusions. To advance the field, gathering a more international perspective may be warranted.

In summary, this project provides an overview of the evidence related to KT in cancer control. Given the current state of the evidence and the need for additional research in so many areas, we were not able to offer a definitive blueprint outlining a small manageable set of research priorities in this context. The field is open and considerable work is required. To ensure world-class research and research that will have a positive impact on people with cancer and on cancer system performance requires clarity and transparency of research scope and goals coupled with high expectations for the research community to achieve excellence in study design, execution, and reporting.

## Competing interests

The authors declare that they have no competing interests.

## Authors' contributions

MCB conceived of the study and participated in its design and execution. JM participated in the study's design and coordination. MCB, JM, and KG were directly involved in the preparation of this manuscript. KG and LD were responsible for data collection and data synthesis. All authors read and approved the final manuscript.

## Endnotes

^a^Interventions to influence uptake of cancer screening were explored in a separate project and were not considered here [17]. Cancer prevention was out of scope for this project and the aforementioned project.

^b^One eligible systematic review by Grimshaw *et al. *(2006) was inadvertently deleted from the systematic review sample [19]. It focused on guidance dissemination and implementation strategies. They found absolute improvement in performance of 14.1% for reminders, 8.1% for dissemination, 7.0% for audit and feedback, and 6% for multifaceted interventions. This review is not included in the summary statistics presented.

## Supplementary Material

Additional file 1 **Research Team**. List of the Research Team members and their affiliations Click here for file

Additional file 2 **Eligible Systematic Reviews**. List of full citations of systematic reviews and the corresponding intervention cluster(s) [23-56] Click here for file

Additional file 3 **Interventions aimed at consumers**. Table of data on each intervention aimed at consumers Click here for file

Additional file 4 **Interventions aimed at professionals**. Table of data on each intervention aimed at professionals Click here for file

Additional file 5 **Interventions aimed at organizations**. Table of data on each intervention aimed at organizations Click here for file

## References

[B1] Mandel JS Bond JH Church TR Snover DC Bradley GM Schuman LM Ederer F Reducing mortality from colorectal cancer by screening for fecal occult blood N Engl J Med 1993 328 1365 1371 10.1056/NEJM199305133281901 8474513

[B2] Hardcastle JD Chamberlain JO Robinson MH Moss SM Amar SS Balfour TW James PD Mangham CM Randomised controlled trial of faecal-occult-blood screening for colorectal cancer Lancet 1996 348 1472 1477 10.1016/S0140-6736(96)03386-7 8942775

[B3] Kronborg O Fenger C Olsen J Jørgensen OD Søndergaard O Randomised study of screening for colorectal cancer with faecal-occult-blood test Lancet 1996 348 1467 1471 10.1016/S0140-6736(96)03430-7 8942774

[B4] Mandel JS Church TR Bond JH Ederer F Geisser MS Mongin SJ Snover DC Schuman LM The effect of fecal occult-blood screening on the incidence of colorectal cancer N Engl J Med 2000 343 1603 1607 10.1056/NEJM200011303432203 11096167

[B5] Verma S Trudeau M Pritchard K Oliver T members of the Breast Cancer Disease Site Group The role of the taxanes in the management of metastatic breast cancer http://www.cancercare.on.ca/common/pages/UserFile.aspx?fileId=34140 Practice Guideline Report # 1-3 Version 2.2003.

[B6] Trudeau M Eisen A Messersmith H Pritchard KI and the Breast Cancer Disease Site Group Adjuvant taxane therapy for women with early-stage, invasive breast cancer: A Clinical Practice Guideline 2006 http://www.cancercare.on.ca/common/pages/UserFile.aspx?fileId=34128 Evidence-based Series #1-7.

[B7] Trudeau M Sinclair S Clemons M Shelley W members of the Breast Cancer Disease Site Group The role of taxanes in neoadjuvant chemotherapy for women with non-metastatic breast cancer 2004 http://www.cancercare.on.ca/common/pages/UserFile.aspx?fileId=13886 Practice Guideline Report #1-20,

[B8] Crump M Trudeau M Sinclair S O'Malley F members of the Breast Cancer Disease Site Group The role of trastuzumab (Herceptin^®^) in the treatment of women with HER2/*neu*-overexpressing metastatic breast cancer http://www.cancercare.on.ca/common/pages/UserFile.aspx?fileId=13870 Practice Guideline Report #1-15 (Version 2.2004)

[B9] Hanks G Cherny N Fallon M Doyle D, Hanks G, Cherny N and Calman K Opioid analgesic therapy Oxford Textbook of Palliative Medicine 2005 3 New York: Oxford University Press 318

[B10] Francke AL Smit MC de Veer AJE Mistiaen P Factors influencing the implementation of clinical guidelines for health care professionals: a systematic meta-review BMC Med Inform Decis Mak 2008 8 38 10.1186/1472-6947-8-38 18789150 PMC2551591

[B11] Cancer Quality Council of Ontario Cancer Quality Index of Ontario 2009 2010 http://csqi.cancercare.on.ca/cms/One.aspx?portalId=40955&pageId=41027

[B12] Graham ID Logan J Harrison MB Straus SE Tetroe J Caswell W Robinson N Lost in knowledge translation: Time for a map? J Contin Educ Health Prof 2006 26 13 24 10.1002/chp.47 16557505

[B13] Dobrow MJ Goel V Lemieux-Charles L Black NA The impact of context on evidence utilization: a framework for expert groups developing health policy recommendations Soc Sci Med 2006 63 1811 1824 10.1016/j.socscimed.2006.04.020 16764980

[B14] Kitson A Rycroft-Malone J Harvey G McCormack B Seers K Titchen A Evaluating the successful implementation of evidence into practice using the PARIHS framework: theoretical and practical challenges Implement Sci 2008 3 1 10.1186/1748-5908-3-1 18179688 PMC2235887

[B15] Ward V House A Hamer S Developing a framework for transferring knowledge into action: a thematic analysis of the literature J Health Serv Res Policy 2009 14 156 164 10.1258/jhsrp.2009.008120 19541874 PMC2933505

[B16] Shojania KG Sampson M Ansari MB Ji J Doucette S Moher D How quickly do systematic reviews go out of date? A survival analysis Ann Intern Med 2007 21 224 233 10.7326/0003-4819-147-4-200708210-00179 17638714

[B17] Brouwers M De Vito C Carol A Carroll J Cotterchio M Dobbin M Lent B Levitt C Lewis N McGregor SE Paszat L Rand C Wathen N Interventions to increase the uptake of cancer screening: guideline recommendations 2009 http://www.cancercare.on.ca/common/pages/UserFile.aspx?fileId=43168 Special Report EBS 15-7.

[B18] Shea BJ Grimshaw JM Wells GA Boers M Andersson N Hamel C Porter AC Tugwell P Moher D Bouter LM Development of AMSTAR: a measurement tool to assess the methodological quality of systematic reviews BMC Med Res Methodol 2007 7 10 10.1186/1471-2288-7-10 17302989 PMC1810543

[B19] Grimshaw J Eccles M Thomas R MacLennan G Ramsay C Fraser C Vale L Toward evidence-based quality improvement. Evidence (and its limitations) of the effectiveness of guideline dissemination and implementation strategies 1966-1998 J Gen Intern Med 2006 21 S14 20 16637955 10.1111/j.1525-1497.2006.00357.x PMC2557130

[B20] Michie S Designing and implementing behavior change interventions to improve population health J Health Serv Res Policy 2008 13 64 69 10.1258/jhsrp.2008.008014 18806194

[B21] Abraham C Michie S A taxonomy of behavior change techniques used in interventions Health Psychol 2008 27 379 387 18624603 10.1037/0278-6133.27.3.379

[B22] Cochrane Effective Practice and Organisation of Care Group (EPOC) (2011) The data collection checklist 2011 http://www.epoc.cochrane.org/

[B23] Ammenwerth E Schnell-Inderst P Machan C Siebert U The effect of electronic prescribing on medication errors and adverse drug events: A systematic review J Am Med Inform Assoc 2008 15 585 600 10.1197/jamia.M2667 18579832 PMC2528040

[B24] Baker R Camosso-Stefinovic J Gillies C Shaw EJ Cheater F Flottorp S Robertson N Tailored interventions to overcome identified barriers to change: Effects on professional practice and health care outcomes Cochrane Database Syst Rev 2010 3 CD005470 20238340 10.1002/14651858.CD005470.pub2 PMC4164371

[B25] Beach MC Gary TL Price EG Robinson K Gozu A Palacio A Smarth C Jenckes M Feuerstein C Bass EB Powe NR Cooper LA Improving health care quality for racial/ethnic minorities: A systematic review of the best evidence regarding provider and organization interventions BMC Public Health 2006 6 104 10.1186/1471-2458-6-104 16635262 PMC1525173

[B26] Bennett MI Bagnall AM José Closs S How effective are patient-based educational interventions in the management of cancer pain? Systematic review and meta-analysis Pain 2009 143 192 199 10.1016/j.pain.2009.01.016 19285376

[B27] Bosch-Capblanch X Abba K Prictor M Garner P Contracts between patients and healthcare practitioners for improving patients' adherence to treatment, prevention and health promotion activities Cochrane Database Syst Rev 2007 18 CD004808 10.1002/14651858.CD004808.pub3 PMC6464838 17443556

[B28] Chaudhry B Wang J Wu S Maglione M Mojica W Roth E Morton SC Shekelle PG Systematic review: impact of health information technology on quality, efficiency, and costs of medical care Ann Intern Med 2006 144 742 752 16702590 10.7326/0003-4819-144-10-200605160-00125

[B29] Coleman K Austin BT Brach C Wagner EH Evidence on the chronic care model in the new millennium Health Aff (Millwood) 2009 28 75 85 10.1377/hlthaff.28.1.75 PMC5091929 19124857

[B30] Conn VS Hafdahl AR Brown SA Brown LM Meta-analysis of patient education interventions to increase physical activity among chronically ill adults Patient Educ Couns 2008 70 157 172 10.1016/j.pec.2007.10.004 18023128 PMC2324068

[B31] Doumit G Gattellari M Grimshaw J O'Brien MA Local opinion leaders: Effects on professional practice and health care outcomes Cochrane Database Syst Rev 2007 1 CD000125 17253445 10.1002/14651858.CD000125.pub3

[B32] Edwards A Gray J Clarke A Dundon J Elwyn G Gaff C Hood K Iredale R Sivell S Shaw C Thornton H Interventions to improve risk communication in clinical genetics: systematic review Patient Educ Couns 2008 71 4 25 10.1016/j.pec.2007.11.026 18207694

[B33] Evans R Edwards A Brett J Bradburn M Watson E Austoker J Elwyn G Reduction in uptake of PSA tests following decision aids: systematic review of current aids and their evaluations Patient Educ Couns 2005 58 13 26 10.1016/j.pec.2004.06.009 15950832

[B34] Garg AX Adhikari NK McDonald H Rosas-Arellano MP Devereaux PJ Beyene J Sam J Haynes RB Effects of computerized clinical decision support systems on practitioner performance and patient outcomes: a systematic review JAMA 2005 293 1223 1238 10.1001/jama.293.10.1223 15755945

[B35] Gaston CM Mitchell G Information giving and decision-making in patients with advanced cancer: a systematic review Soc Sci Med 2005 61 2252 2264 10.1016/j.socscimed.2005.04.015 15922501

[B36] Goldberg GR Morrison RS Pain management in hospitalized cancer patients: a systematic review J Clin Oncol 2007 25 1792 1801 10.1200/JCO.2006.07.9038 17470871

[B37] Gysels M Richardson A Higginson IJ Does the patient-held record improve continuity and related outcomes in cancer care: a systematic review Health Expect 2007 10 75 91 10.1111/j.1369-7625.2006.00415.x 17324196 PMC5060382

[B38] Haynes RB Ackloo E Sahota N McDonald HP Yao X Interventions for enhancing medication adherence Cochrane Database Syst Rev 2008 2 CD000011 18425859 10.1002/14651858.CD000011.pub3

[B39] Heneghan CJ Glasziou P Perera R Reminder packaging for improving adherence to self-administered long-term medications Cochrane Database Syst Rev 2006 1 CD005025 16437510 10.1002/14651858.CD005025.pub2

[B40] Jamtvedt G Young JM Kristoffersen DT O'Brien MA Oxman AD Audit and feedback: effects on professional practice and health care outcomes Cochrane Database Syst Rev 2006 2 CD000259 16625533 10.1002/14651858.CD000259.pub2

[B41] Joosten EA DeFuentes-Merillas L de Weert GH Sensky T van der Staak CP de Jong CA Systematic review of the effects of shared decision-making on patient satisfaction, treatment adherence and health status Psychother Psychosom 2008 77 219 226 10.1159/000126073 18418028

[B42] Lewis RA Neal RD Williams NH France B Hendry M Russell D Hughes DA Russell I Stuart NS Weller D Wilkinson C Follow-up of cancer in primary care versus secondary care: systematic review Br J Gen Pract 2009 59 e234 47 19566990 10.3399/bjgp09X453567 PMC2702037

[B43] Murray E Burns J See TS Lai R Nazareth I Interactive health communication applications for people with chronic disease Cochrane Database Syst Rev 2005 4 CD004274 16235356 10.1002/14651858.CD004274.pub4 PMC13184810

[B44] O'Brien MA Rogers S Jamtvedt G Oxman AD Odgaard-Jensen J Kristoffersen DT Forsetlund L Bainbridge D Freemantle N Davis DA Haynes RB Harvey EL Educational outreach visits: effects on professional practice and health care outcomes Cochrane Database Syst Rev 2007 4 CD000409 17943742 10.1002/14651858.CD000409.pub2 PMC7032679

[B45] Raynor DK Blenkinsopp A Knapp P Grime J Nicolson DJ Pollock K Dorer G Gilbody S Dickinson D Maule AJ Spoor P A systematic review of quantitative and qualitative research on the role and effectiveness of written information available to patients about individual medicines Health Technol Assess 2007 11 iii, 1-160. 10.3310/hta11050 17280623

[B46] Rotter T Kinsman L James E Machotta A Willis J Snow P Kugler J Clinical pathways: Effects on professional practice, patient outcomes, length of stay and hospital costs Cochrane Database Syst Rev 2010 3 CD006632 20238347 10.1002/14651858.CD006632.pub2

[B47] Santo A Laizner AM Shohet L Exploring the value of audiotapes for health literacy: a systematic review Patient Educ Couns 2005 58 235 243 10.1016/j.pec.2004.07.001 16054796

[B48] Scheuner MT Sieverding P Shekelle PG Delivery of genomic medicine for common chronic adult diseases: a systematic review JAMA 2008 299 1320 1334 10.1001/jama.299.11.1320 18349093

[B49] Shepperd S McClaran J Phillips CO Lannin NA Clemson LM McCluskey A Cameron ID Barras SL Discharge planning from hospital to home Cochrane Database Syst Rev 2010 1 CD000313 20091507 10.1002/14651858.CD000313.pub3

[B50] Shojania KG Jennings A Mayhew A Ramsay CR Eccles MP Grimshaw J The effects of on-screen, point of care computer reminders on processes and outcomes of care Cochrane Database Syst Rev 2009 3 CD001096 19588323 10.1002/14651858.CD001096.pub2 PMC4171964

[B51] Smith SM Allwright S O'Dowd T Effectiveness of shared care across the interface between primary and specialty care in chronic disease management Cochrane Database Syst Rev 2007 3 CD004910 17636778 10.1002/14651858.CD004910.pub2

[B52] Smith SM Allwright S O'Dowd T Does sharing care across the primary-specialty interface improve outcomes in chronic disease? a systematic review Am J Manag Care 2008 14 213 224 18402514

[B53] Thomas L Cullum N McColl E Rousseau N Soutter J Steen N Guidelines in professions allied to medicine Cochrane Database Syst Rev 2000 2 CD000349 10796531 10.1002/14651858.CD000349 PMC13110913

[B54] Waljee JF Rogers MA Alderman AK Decision aids and breast cancer: Do they influence choice for surgery and knowledge of treatment options? J Clin Oncol 2007 25 1067 73 10.1200/JCO.2006.08.5472 17369570

[B55] Wilson AD Childs S Effects of interventions aimed at changing the length of primary care physicians' consultation Cochrane Database Syst Rev 2006 1 CD003540 16437458 10.1002/14651858.CD003540.pub2

[B56] Wofford JL Smith ED Miller DP The multimedia computer for office-based patient education: a systematic review Patient Educ Couns 2005 2 148 57 10.1016/j.pec.2004.10.011 16257619

