# Supplementary material for: The landscape of knowledge translation interventions in cancer control: What do we know and where to next? A review of systematic reviews
Source: Implement Sci. 2011 Dec 20;6:130. doi: 10.1186/1748-5908-6-130 (PMC3284444; doi:10.1186/1748-5908-6-130)
Supplement: Additional file 1 — Research Team. List of the Research Team members and their affiliations [file 1748-5908-6-130-S1.DOC]

**Additional file 1:** **Research Team**

| **Team Member** | **Affiliations** |
| --- | --- |
| **Brouwers, Melissa** | - Associate Professor and Lead of Health Services Research, McMaster University, Department of Oncology - Associate Member, McMaster University, Department of Clinical Epidemiology and Biostatistics - National Lead, Capacity Enhancement Program, Canadian Partnership Against Cancer - Provincial Director, Program in Evidence-based Care, Cancer Care Ontario |
| **Browman, George** | - Medical Oncologist, British Columbia Cancer Agency - Professor (PT), McMaster University, Department of Clinical Epidemiology and Biostatistics - Chair, Cancer Guidelines Advisory Group, Canadian Partnership Against Cancer |
| **Bryant-Lukosius,**  **Denise** | - Assistant Professor, McMaster University, School of Nursing and Associate Member, Department of Oncology - Director, Canadian Centre of Excellence in Oncology Advanced Practice Nursing (OAPN) |
| **Dobbins, Maureen** | - Associate Professor, McMaster University, Department of Nursing - Associate Member, McMaster University, Department of Clinical Epidemiology and Biostatistics and School of Rehabilitation Science - Scientific Director of Health-Evidence.ca |
| **Elit, Laurie** | - Gynecology Oncologist, Juravinski Cancer Centre - Associate Professor, McMaster University, Department Ob-Gyn |
| **Esmail, Rosmin** | - Director, Leading Practices, Knowledge Management, Alberta Health Services |
| **Gagliardi, Anna** | - Assistant Professor, University of Toronto, Department of Surgery; and Health Policy, Evaluation and Management and Institute of Medical Science, Faculty of Medicine - Affiliate Scientist, Toronto General Research Institute |
| **Graham, Ian** | - Vice-President, Knowledge Translation and Commercialization, Canadian Institutes for Health Research |
| **Grimshaw, Jeremy** | - Director, Clinical Epidemiology Program, Ottawa Health Research Institute - Coordinating Editor, EPOC, Cochrane Collaboration - Co-PI, KT Canada |
| **Hagen, Neil** | - Neurological Oncologist, Alberta Health Services, Cancer Care - Medical Leader, Guideline Utilization Resource Unit, Alberta Health Services Cancer Care - Interim Director, Provincial Clinical Teams, Alberta Health Services - Member, Cancer Guidelines Advisory Group, Canadian Partnership Against Cancer |
| **Schünemann, Holger** | - Internist, Hamilton Health Sciences Centre - Professor and Chair, McMaster University, Department of Clinical Epidemiology and Biostatistics |
| **Levine,**  **Mark** | - Medical Oncologist, Juravinski Cancer Centre - Professor and Chair, McMaster University, Department of Oncology |
| **Levitt,**  **Cheryl** | - Professor, McMaster University, Department of Family Medicine - Provincial Lead, Primary Care, Cancer Care Ontario |
| **Pasut,**  **George** | - Vice President, Science and Public Health, Ontario Agency for Health Protection and Promotion |
| **Petrella,**  **Jill** | - Manager, Cancer Care Nova Scotia, Quality and Guidelines Initiative - Member, Atlantic Provinces Cancer Guideline Collaborative - Member, Cancer Guidelines Advisory Group, Canadian Partnership Against Cancer |
| **Rand,**  **Carol** | - Director, Systemic, Supportive and Regional Cancer Programs, Juravinski Cancer Centre - Clinical Instructor, McMaster University |
| **Sawka,**  **Carol** | - Medical Oncologist, Odette Cancer Centre - Vice President, Clinical Programs & Quality Initiatives, Cancer Care Ontario - Professor, University of Toronto, Departments of Medicine, Public Health Sciences, Health Policy Management and Evaluation |
| **Seow,**  **Hsien** | - Assistant Professor, McMaster University, Department of Oncology - Associate Member, McMaster University, Department of Clinical Epidemiology and Biostatistics |
| **Simunovic, Marko** | - Surgical Oncologist, Juravinski Cancer Centre – Hamilton Health Sciences - Associate Professor, McMaster University, Department of Surgery - Associate Member, McMaster University, Department of Clinical Epidemiology and Biostatistics |
| **Straus,**  **Sharon** | - Professor, University of Toronto, Department of Medicine - Division Director, Division of Geriatric Medicine, Department of Medicine, University of Toronto - Scientist, St. Michael’s Hospital, Keenan Research Centre of the Li Ka Shing Knowledge Institute - Director, Knowledge Translation Program, Li Ka Shing Knowledge Institute at St. Michael’s Hospital and University of Toronto - Co-PI, KT Canada |
| **Sussman,**  **Jonathan** | - Radiation Oncologist, Juravinski Cancer Centre - Associate Professor, McMaster University, Department of Oncology - Director, Supportive Cancer Care Research Unit, Juravinski Cancer Centre |
