# Supplementary material for: The landscape of knowledge translation interventions in cancer control: What do we know and where to next? A review of systematic reviews
Source: Implement Sci. 2011 Dec 20;6:130. doi: 10.1186/1748-5908-6-130 (PMC3284444; doi:10.1186/1748-5908-6-130)
Supplement: Additional file 2 — Eligible Systematic Reviews. List of full citations of systematic reviews and the corresponding intervention cluster(s) [23-56] [file 1748-5908-6-130-S2.DOC]

**Additional file 2. Eligible Systematic Reviews**

| **Full citation** | **Intervention Cluster** |
| --- | --- |
| Ammenwerth E, Schnell-Inderst P, Machan C, Siebert U: **The effect of electronic prescribing on medication errors and adverse drug events: A systematic review**. *J Am Med Inform Assoc* 2008, **15**:585-600. [23] | Professional |
| Baker R, Camosso-Stefinovic J, Gillies C, Shaw EJ, Cheater F, Flottorp S, Robertson N: **Tailored interventions to overcome identified barriers to change: Effects on professional practice and health care outcomes**. *Cochrane Database Syst Rev* 2010, **3**:CD005470. [24] | Professional  Organizational |
| Beach MC, Gary TL, Price EG, Robinson K, Gozu A, Palacio A, Smarth C, Jenckes M, Feuerstein C, Bass EB, Powe NR, Cooper LA: **Improving health care quality for racial/ethnic minorities: A systematic review of the best evidence regarding provider and organization interventions**. *BMC Public Health* 2006, **6**:104. [25] | Professional  Organizational |
| Bennett MI, Bagnall AM, José Closs S: **How effective are patient-based educational interventions in the management of cancer pain? Systematic review and meta-analysis**. *Pain* 2009, **143**(3):192-199. [26] | Consumer |
| Bosch-Capblanch X, Abba K, Prictor M, Garner P: **Contracts between patients and healthcare practitioners for improving patients' adherence to treatment, prevention and health promotion activities**. *Cochrane Database Syst Rev* 2007, **18**:CD004808. [27] | Consumer |
| Chaudhry B, Wang J, Wu S, Maglione M, Mojica W, Roth E, Morton SC, Shekelle PG: **Systematic review: impact of health information technology on quality, efficiency, and costs of medical care**. *Ann Intern Med* 2006, **144**:742-752. [28] | Organizational |
| Coleman K, Austin BT, Brach C, Wagner EH: **Evidence on the chronic care model in the new millennium**. *Health Aff (Millwood)* 2009, **28**:75-85. [29] | Organizational |
| Conn VS, Hafdahl AR, Brown SA, Brown LM: **Meta-analysis of patient education interventions to increase physical activity among chronically ill adults**. *Patient Educ Couns* 2008, **70**:157-172. [30] | Consumer |
| Doumit G, Gattellari M, Grimshaw J, O'Brien MA: Local opinion leaders: **Effects on professional practice and health care outcomes**. *Cochrane Database Syst Rev* 2007, **1**:CD000125. [31] | Professional |
| Edwards A, Gray J, Clarke A, Dundon J, Elwyn G, Gaff C, Hood K, Iredale R, Sivell S, Shaw C, Thornton H: **Interventions to improve risk communication in clinical genetics: systematic review**. *Patient Educ Couns* 2008, **71**:4-25. [32] | Consumer |
| Evans R, Edwards A, Brett J, Bradburn M, Watson E, Austoker J, Elwyn G: **Reduction in uptake of PSA tests following decision aids: systematic review of current aids and their evaluations**. *Patient Educ Couns* 2005, **58**:13-26. [33] | Consumer |
| Garg AX, Adhikari NK, McDonald H, Rosas-Arellano MP, Devereaux PJ, Beyene J, Sam J, Haynes RB: **Effects of computerized clinical decision support systems on practitioner performance and patient outcomes: a systematic review**. *JAMA* 2005, **293**:1223-1238. [34] | Professional |
| Gaston CM, Mitchell G: **Information giving and decision-making in patients with advanced cancer: a systematic review**. *Soc Sci Med* 2005, **61**:2252-2264. [35] | Consumer |
| Goldberg GR, Morrison RS: **Pain management in hospitalized cancer patients: a systematic review**. *J Clin Oncol* 2007, **25**:1792-1801. [36] | Professional  Consumer  Organizational |
| Gysels M, Richardson A, Higginson IJ: **Does the patient-held record improve continuity and related outcomes in cancer care: a systematic review**. *Health Expect* 2007, **10**:75-91. [37] | Consumer |
| Haynes RB, Ackloo E, Sahota N, McDonald HP, Yao X: **Interventions for enhancing medication adherence**. *Cochrane Database Syst Rev* 2008, **2**:CD000011. [38] | Consumer |
| Heneghan CJ, Glasziou P, Perera R: **Reminder packaging for improving adherence to self-administered long-term medications**. *Cochrane Database Syst Rev* 2006, **1**:CD005025. [39] | Consumer |
| Jamtvedt G, Young JM, Kristoffersen DT, O'Brien MA, Oxman AD: **Audit and feedback: effects on professional practice and health care outcomes**. *Cochrane Database Syst Rev* 2006, **2**:CD000259. [40] | Professional |
| Joosten EA, DeFuentes-Merillas L, de Weert GH, Sensky T, van der Staak CP, de Jong CA: **Systematic review of the effects of shared decision-making on patient satisfaction, treatment adherence and health status**. *Psychother Psychosom* 2008, **77**:219-226. [41] | Consumer |
| Lewis RA, Neal RD, Williams NH, France B, Hendry M, Russell D, Hughes DA, Russell I, Stuart NS, Weller D, Wilkinson C: **Follow-up of cancer in primary care versus secondary care: systematic review**. *Br J Gen Pract* 2009, **59**:e234-47. [42] | Organizational |
| Murray E, Burns J, See TS, Lai R, Nazareth I: **Interactive health communication applications for people with chronic disease**. *Cochrane Database Syst Rev* 2005, **(4)**:CD004274. [43] | Consumer |
| O'Brien MA, Rogers S, Jamtvedt G, Oxman AD, Odgaard-Jensen J, Kristoffersen DT, Forsetlund L, Bainbridge D, Freemantle N, Davis DA, Haynes RB, Harvey EL: **Educational outreach visits: effects on professional practice and health care outcomes**. *Cochrane Database Syst Rev* 2007, **(4)**:CD000409. [44] | Professional |
| Raynor DK, Blenkinsopp A, Knapp P, Grime J, Nicolson DJ, Pollock K, Dorer G, Gilbody S, Dickinson D, Maule AJ, Spoor P: **A systematic review of quantitative and qualitative research on the role and effectiveness of written information available to patients about individual medicines**. *Health Technol Assess* 2007, **11**:iii,1-160. [45] | Consumer |
| Rotter T, Kinsman L, James E, Machotta A, Willis J, Snow P, Kugler J: **Clinical pathways: Effects on professional practice, patient outcomes, length of stay and hospital costs**. *Cochrane Database Syst Rev* 2010, **3**:CD006632. [46]^a^ | Professional |
| Santo A, Laizner AM, Shohet L: **Exploring the value of audiotapes for health literacy: a systematic review**. *Patient Educ Couns* 2005, **58**:235-243. [47] | Consumer |
| Scheuner MT, Sieverding P, Shekelle PG: **Delivery of genomic medicine for common chronic adult diseases: a systematic review**. *JAMA* 2008, **299**:1320-1334. [48] | Organizational |
| Shepperd S, McClaran J, Phillips CO, Lannin NA, Clemson LM, McCluskey A, Cameron ID, Barras SL: **Discharge planning from hospital to home**. *Cochrane Database Syst Rev* 2010, **(1)**:CD000313. [49]^a^ | Professional |
| Shojania KG, Jennings A, Mayhew A, Ramsay CR, Eccles MP, Grimshaw J: **The effects of on-screen, point of care computer reminders on processes and outcomes of care**. *Cochrane Database Syst Rev* 2009, **(3)**:CD001096. [50] | Professional |
| Smith SM, Allwright S, O'Dowd T: **Effectiveness of shared care across the interface between primary and specialty care in chronic disease management**. *Cochrane Database Syst Rev* 2007, **(3)**:CD004910. [51] | Organizational |
| Smith SM, Allwright S, O'Dowd T: **Does sharing care across the primary-specialty interface improve outcomes in chronic disease? a systematic review**. *Am J Manag Care* 2008, **14**:213-224. [52] | Organizational |
| Thomas L, Cullum N, McColl E, Rousseau N, Soutter J, Steen N: **Guidelines in professions allied to medicine**. *Cochrane Database Syst Rev* 2000, **(2)**:CD000349. [53]^a^ | Professional |
| Waljee JF, Rogers MA, Alderman AK: **Decision aids and breast cancer: Do they influence choice for surgery and knowledge of treatment options?** *J Clin Oncol* 2007, **25**:1067-73. [54] | Consumer |
| Wilson AD, Childs S: **Effects of interventions aimed at changing the length of primary care physicians’ consultation**. *Cochrane Database Syst Rev* 2006, **(1)**:CD003540. [55] | Organizational |
| Wofford JL, Smith ED, Miller DP: **The multimedia computer for office-based patient education: a systematic review**. *Patient Educ Couns* 2005, **(2)**:148-57. [56] | Consumer |

^a^These reviews were included *post hoc*, identified by members of the research team.
