# Supplementary material for: The landscape of knowledge translation interventions in cancer control: What do we know and where to next? A review of systematic reviews
Source: Implement Sci. 2011 Dec 20;6:130. doi: 10.1186/1748-5908-6-130 (PMC3284444; doi:10.1186/1748-5908-6-130)
Supplement: Additional file 3 — Interventions aimed at consumers. Table of data on each intervention aimed at consumers [file 1748-5908-6-130-S3.DOC]

Additional file 3: Interventions aimed at consumers

| **SR 1^st^ Author (Year)** | **Title of SR** | **Study designs in SR (#)** | **Intervention details** | **Results** |
| --- | --- | --- | --- | --- |
| **Patient Education/Patient Information, n = 8** | | | | |
| Bennett (2009) [26] | How effective are patient-based educational interventions in the management of cancer pain? Systematic review and meta analysis | RCT (19) | - improve management of cancer pain - cancer only - participants – nurse delivered - pain management education using various modalities | - effects on knowledge or attitudes (vs. control)   - improve 0.5 on 5 point scale (WMD = -0.52, CI 0.04 to 1.0) - effects on median pain intensity (vs. control)   - reduction of 1 on 10 point scale (WMD= -1.1, CI -1.8 to -0.41) - effect on maximum pain intensity   - reduction of 0.78 on 10 point scale (WMD= -0.78, CI -1.21 to -0.35) |
| Gaston (2005) [35] | Information giving and decision-making in patients with advanced cancer: a systematic review | RCT (12)  DS (22)  non-RCT (10)  RT (3) | - improve knowledge, satisfaction and decision making - cancer only - participants – physicians and other health care providers - various educational interventions considered including provision of consultation tapes, question prompt sheets, decision aids | - knowledge and satisfaction results   - strategies had some effect on patient knowledge and satisfaction with clinical encounter but little evidence of impact on patient psychological outcomes - general   - patient-specific info yields greater satisfaction than general info |
| Gysels (2006) [37] | Does the patient held record improve continuity and related outcomes in cancer care: a systematic review | RCT (7)  non-RCT (7) | - improve end-of-life care - cancer only - participants – patients and nurses - patient held paper medical record (various forms: log book, client record) to complement medical record | - no impact on information seeking, retention, or understanding - satisfaction: 1 of 6 trials show favourable impact on satisfaction with communication, 4 show no effect, and 1 shows less satisfaction with information - attitudes: no difference in patients’ perceptions of how well they are informed - general   - studies consistently fail to show positive results in favour of PHR   - PHR are perceived as useful by clinical care team but little actual use |
| Goldberg (2007) [36] | Pain Management in Hospitalized Cancer Patients: A Systematic Review | RCT (3)  pre-post (3) | - improve patient management - cancer only - participants – nurses and patients - various educational strategies | - knowledge   - increase in patient and nurse knowledge about pain - satisfaction   - no increase in patient satisfaction but increase nurse satisfaction - pain and attitudes   - some evidence to suggest some improvements in pain scores and reduction in negative pain beliefs and misconceptions |
| Wofford (2005) [56] | The multimedia computer for office-based patient education: a systematic review | RCT (26) | - impact on knowledge, attitudes, clinical outcomes, and behavioral compliance - participants – patients, physicians, providers - mixed population with cancer - multimedia educational strategies (animation, video, audio) in office environments | - knowledge: 9 of 10 studies found improvement in patient knowledge - attitudes: 2 of 3 studies found improvements in patient attitudes - clinical outcomes   - 5 of 14 studies found improvement on some clinical outcomes   - 8 of 14 studies found no difference   - 1 study reported greater anxiety in experimental group - behavioral compliance: 8 of 9 studies found positive impact on compliance - general   - multimedia educational interventions hold promise   - ability to customize to specifics of patients will be important |
| Santo (2005) [47] | Exploring the value of audiotapes for health literacy: a systematic review | RCT (17)  non-RCT (1)  TA (1)  surveys (9)  DS (4),  LR (3) | - impact on knowledge, satisfaction, and behavioral compliance - participants - patients - mixed population with cancer - provision of audiotaped recordings of consultation with patients | - knowledge   - 9 studies found improvement in patient knowledge   - 1 study found reduced knowledge with general information tape   - 3 studies found no difference - satisfaction   - 4 studies found improved patient satisfaction with consultation tape versus general information tape   - 2 studies found no difference - behavioral compliance   - 4 studies found positive behavior change (3 statistically significant)   - 1 study found no positive correlation - general   - consultation tape interventions hold promise |
| Conn (2008) [30] | Meta-Analysis of Patient Education Interventions to Increase Physical Activity among Chronically Ill Adult | Total: 163 | - to increase physical activity among patients - participants - patients - mixed population with cancer - patient education (mixed methods – practice exercises, behavioral modification) | - smallest effect size seen with cancer patients - less complex interventions were as effective as complex interventions |
| Raynor (2007) [45] | A systematic review of quantitative and qualitative research on the role and effectiveness of written information available to patients about individual medicines | RCT (7)  MM (63) | - to improve outcomes and patient safety - participants – patients, physicians, nurses - mixed population with cancer - provision of written patient education materials regarding medication and medication use | - knowledge   - 8 of 33 studies found improvement in patient knowledge   - 4 of 33 studies found reduced patient knowledge   - 21 of 33 studies found no difference - satisfaction   - impact depended on information presented with numeric descriptions yielding greater satisfaction than text descriptions - attitudes and intentions to behave   - impact depended on information presented with numeric descriptions yielding greater intentions to comply than text descriptions - general   - written materials are often of poor quality and not accessible   - written materials often not valued |
| **Patient Decision Aids, n = 5** | | | | |
| Evans (2005) [33] | Reduction in uptake of PSA tests following decision aids: systematic review of current aids and their evaluations | RCT (8)  non-RCT (2)  survey (1)  2x2 FC (1)  NS (31) | - impact on PSA uptake, PSA knowledge - cancer context only - men - participants – patients, primary care physicians - mixed formats and modalities (paper, electronic, static, interactive) | - reduced probability in PSA testing after a decision aid: -3.5%, p=0.050 - increased knowledge: 19.5%, p<0.001 - sustained knowledge 12-18 months after a decision aid: 3.4%, p=0.10 |
| Gaston (2005) [35] | Information giving and decision-making in patients with advanced cancer: a systematic review | DS (22)  RCT (12)  non-RCT (10)  RT (3) | - impact on end of life care - cancer patients only - participants – patients, physicians, nurses, other health care providers - more of a scoping review that considered decision making in broadest sense | - tools such as decision aids, that support decision making may be reasonable options for some patients |
| Waljee (2007) [54] | Decision Aids and Breast Cancer: Do They Influence Choice for Surgery and Knowledge of Treatment Options? | RCT (3)  non-RCT (8) | - impact of decision aids for surgical treatment of early breast cancer - cancer patients only - participants – patients and physicians - mixed formats and modalities | - behaviour: patients 25% more likely to choose breast-conserving surgery over mastectomy (RR = 1.25, 95% CI 1.11 to 1.40) - knowledge: increased by 24% (p=0.024) - decisional conflict: reduced - satisfaction: increased |
| Edwards (2008) [32] | Interventions to improve risk communication in clinical genetics: Systematic review. | RCT (6)  other (6) | - impact of decision aids on knowledge of clinical genetics - mixed population with cancer - participants – patients, physicians, other health care providers, nurses - considered several interventions including decision aids | - improve knowledge - improve perceptions of genetic risk |
| Joosten (2008) [41] | Systematic Review of the Effects of Shared Decision-Making on Patient Satisfaction Treatment Adherence and Health Status | RCT (17) | - mixed population with cancer - shared decision making whereby providers and patients share preference for treatment and arrive at a treatment choice | - knowledge: 2 studies in favour of SDM, 1 study in favour of control - satisfaction: 1 studies in favour of SDM, 7 studies no difference - well-being (psychological and physical): 2 out 5 studies in favour of SDM - behaviour: 1 study found greater adherence to behavior - overall: 5 studies showed no difference on any of the outcomes |
| **Interactive Health Communication Applications, n = 1** | | | | |
| Murray (2005) [43] | Interactive Health Communication Applications for people with chronic disease | RCT (24) | - mixed population with cancer - participants – patients (adults and children) - IHCA for use by patients and families with engagement by peers or professionals | - increased knowledge (SMD 0.46; 95% confidence interval (CI) 0.22 to 0.69) - improved social support (SMD 0.35; 95% CI 0.18 to 0.52) - improved clinical outcomes (SMD 0.18; 95% CI 0.01 to 0.35) - increased self-efficacy (SMD 0.24; 95% CI 0.00 to 0.48) - improved behavioural outcomes (continuous) (SMD 0.20; 95% CI 0.01 to 0.40) - binary behavioural outcomes (in direction, n.s.; OR 1.66; 95% CI 0.71 to 3.87) |
| **Contracts, n =1** | | | | |
| Bosch-Capblanch (2009) [27] | Contracts between patients and healthcare practitioners for improving patients' adherence to treatment, prevention and health promotion activities | RCT (24) CRCT (6) | - impact on behavior (treatment health promotion activities, etc.), perceptions, harms, costs - mixed population no cancer - participants – providers and patients - multiple formats and modalities, various terms, various incentives | - 15 trials report at least 1 outcome that showed significant differences favoring contracts - 6 trials report at least 1 outcome that showed significant differences favoring control group - 26 trials report at least 1 outcome that shows no difference between groups |
| **Reminder Packaging, n =1** | | | | |
| Heneghan (2008) [39] | Reminder packaging for improving adherence to self-administered long-term medications (Review) | RCT (8) | - effectiveness on patient attitudes, behaviors, and clinical outcomes - mixed population no cancer - participants – patients, pharmacists, other providers - variety of reminder packaging tactics employed | - attitudes   - packaging rated as useful (77% intervention vs. 27% control) - behavior   - significant increase in % of pills taken with reminder package group: WMD 11% (95% CI 6% to 17%) - clinical outcomes   - insufficient evidence |
| **Multifaceted, n = 1** | | | | |
| Haynes (2008) [38] | Interventions for improving medication adherence (Review) | RCT (73)  CRCT (5) | - improve medication adherence - mixed population with cancer (potential) - participants - patients, physicians, nurses, providers, teams, case manager, etc. - variety of clustered delivery modes, formats, technologies, etc (21 descriptive intervention clusters as guides) | - very large number of studies and interventions considered - medication adherence and treatment outcome   - adherence     - 41 of 93 interventions increased medication adherence       - 5 for short-term treatments       - 36 for long-term treatments   - outcome     - 29 of 93 interventions improved treatment outcomes       - 4 for short-term treatments       - 25 for long-term treatments |

NB: SR = systematic review, RCT = randomized controlled trial, DS = descriptive, non-RCT = non-randomized controlled trial, RT = non-controlled randomized trial, TA = tandem assignment, LR = literature review, MM = mixed methods, 2x2 FC = 2x2 factorial comparison, NS = not specified, CRCT = cluster RCT, CCT = controlled clinical trial, TS = time series, PC = prospective cohort, RC = retrospective cohort, CS = cross-sectional, CCCT = cluster controlled clinical trial, QEL = quasi-experimental using linear modeling, CE = cost-effectiveness, RS = relationships, CBA = controlled before-after, OBS = observational, CC = case control
