# Supplementary material for: The landscape of knowledge translation interventions in cancer control: What do we know and where to next? A review of systematic reviews
Source: Implement Sci. 2011 Dec 20;6:130. doi: 10.1186/1748-5908-6-130 (PMC3284444; doi:10.1186/1748-5908-6-130)
Supplement: Additional file 4 — Interventions aimed at professionals. Table of data on each intervention aimed at professionals [file 1748-5908-6-130-S4.DOC]

**Appendix 4**: Interventions aimed at professionals

| **SR 1^st^ Author (Year)** | **Title of SR** | **Study designs in review (#)** | **Intervention details** | **Results** |
| --- | --- | --- | --- | --- |
| **Educational Outreach Visits & Audit and Feedback Interventions, n = 4** | | | | |
| Goldberg (2007) [36] | Pain Management in Hospitalized Cancer Patients: A Systematic Review | RCT (1)  pre-post (3) | - improve management of pain - cancer patients only - participants - nurses - various educational outreach tactics | - increased patient satisfaction - increased documentation - increased nursing knowledge - no improvement with patients’ pain severity ratings |
| O’Brien (2008) [44] | Educational outreach visits and effects on professional practice and health outcomes | RCT (69) | - improve clinical practice (varied practice targeted) - mixed population with cancer - participants - physicians, nurses, other providers - various educational outreach tactics | | Outcomes | EOA part vs None | EOA alone vs None | | --- | --- | --- | | provider practice dichotomous outcomes | | | | ARD median improvement | 5.6%: -3% to 64% | 5.0%: 1% to 20% | | ARD < 5% | 15 of 34 | 9 of 21 | | ARD 5%-9% | 11 of 34 | 8 of 21 | | ARD 10+% | 8 of 34 | 1 of 21 | | provider practice continuous outcomes | | | | A % median improvement | 21%: 0% to 61.4% | 23%: 0% to 617% | | A% <10% | 4 of 20 | - | | A% 11-20% | 5 of 20 | - | | A% 20%+ | 9 of 20 | - | | patient outcomes | | few studies, small effects to no effects | | - EOV at in individual level tend to be more effective than in groups - multifaceted interventions of which EOV is a part tends to be more effective than single interventions - interventions that include EOV tend to be more effective than audit and feedback alone - EOV yields small but consistent benefits for prescribing behavior in contrast to other behaviors where effect sizes vary considerably | | | |
| Goldberg (2007) [36] | Pain Management in Hospitalized Cancer Patients: A Systematic Review | pre-post (3)  CCT (1) | - improve management of pain - cancer patients only - participants - nurses - audit and feedback | - improvements on assessment rates, provider satisfaction but no impact on clinical outcomes (patient pain scores) |
| Jamtvedt (2006) [40] | Audit and feedback: effects on professional practice and health care outcomes | RCT (118) | - improve clinical practice - mixed population with cancer - participants - physicians, nurses, other providers, units, departments, institutions - audit and feedback using various tactics | | Comparison | Provider Practice  dichotomous  ARD improvement | Provider Practice  continuous  A% improvement | | --- | --- | --- | | AF Part vs. None | 5.0%: -16% to 70% | 5.0%: -10% to 68% | | AF Alone vs. None | 4.0%: -16% to 32% | 11.9%: -10.3% to 67.5% | | AF + EOV vs. None | 1.5%: -1% to 24% | 28.7%: 3% to 41% | | AF as part of multifaceted (not EOV) vs. None | 5.7%: -9% to 70% | 23.8%: 3% to 60% | | - very few factors could explain variability of effects - AF may be more effective when baseline compliance is poor though the effect is not large - higher intensity of AF is more effective than lower intensity AF - there is little data to direct how AF should be delivered   - no evidence to support or refute AF more effective with the addition of peer performance data   - no evidence to support or refute AF more effective when provider motivation is high (vs. low or medium)   - AF may be more effective when outcomes are considered serious | | | |
| **IT/IM/Informatic Interventions, n = 5** | | | | |
| Goldberg (2007) [36] | Pain Management in Hospitalized Cancer Patients: A Systematic Review | CCT (1) | - improve management of pain - cancer patients only - participants - physicians - CDSS that provide recommendations to physicians regarding drug options, dose escalation, modifying agents, adverse effects, etc. | - appropriate reduction in prescribing a particular drug - no change in pain scores |
| Garg (2005) [34] | Effects of computerized clinical decision support systems on practitioner performance and patient outcomes: a systematic review | RCT (49) CRCT (38) TS (1)  other (11) | - improve clinical care and patient outcomes across clinical conditions - mixed population with cancer - participants – physicians, interns, residents - interactive CDSS whereby clinical staff or patients enter patient characteristics and receive advice/recommendations | - clinical performance improved in 64% of 97 studies   - 40% using systems targeting diagnosis   - 76% using reminder systems   - 62% using disease management systems   - 66% with using drug dosing and prescribing systems - patient outcomes improved in 13% of 52 studies - general findings - automatic prompt systems greater impact than user activated prompt systems more effective (73% vs. 47% of trials) - systems developed by authors more effective than systems in which authors were not the developers (74% vs. 28% of trials) |
| Ammenwerth (2008) [23] | The effect of electronic prescribing on medication errors and adverse drug events: a systematic review | RCT (1) CRCT (1)  TS (6)  PC (6)  RC (2)  CS (1)  pre-post (9) | - to improve patient safety across clinical conditions - mixed population with cancer - participants – physicians and patients - no decision support (provides a list of drugs, doses, monographs) vs. limited decision support (evidence-based specific recommendations of drugs, doses, etc.) vs. advanced decision support (drug allergy or interaction information, drug-lab alerts, etc.) | - medication error rate   - reduction in error rate for 23/25 studies (RR range 13% to 99%)   - inconclusive findings for 1/25 studies   - increase risk in error rate for 1/25 studies - adverse drug events (potential)   - reduction in adverse drug events for 6/9 studies (RR range 35% to 98%)   - inconclusive findings for 2 of 9 studies   - increase in adverse drug event for 1/9 studies - adverse drug events (actual)   - reduction in adverse drug events for 4/7 studies (RR range 30% to 84%)   - inconclusive finding for 1 of 7 studies   - increase in adverse drug event for 1/7 studies (9%) |
| Beach (2006) [25] | Improving health care quality for racial/ethnic minorities: a systematic review of the best evidence regarding provider and organization interventions | RCT (7)  CCT (3) | - improve quality of health care and reduce disparities - mixed population with cancer - participants – physicians, residents, fellows, nurses - cancer mix | - all studies showed improvements in appropriateness of care (e.g. prevention of preventive care, tobacco cessation counseling, etc.) |
| Shojania (2010) [50] | The effects of on-screen, point of care computer reminders on processes and outcomes of care (review) | RCT (2)  CRCT (20)  CCT (4)  CCCT (2) | - mixed population with cancer - participants – physicians, nurses, pharmacists, dentist - computer system reminders alerting providers to potentially redundant tests | - consistent but small median absolute improvements in process adherence   - across all outcomes 4.2% (IQR 0.8% to 18.8%)   - ordering of medication, 3.3% (IQR 0.5% to 10.6%)   - prescription of vaccines 3.8% (IQR 0.5% to 6.6%)   - test ordering 3.8% (IQR 0.4% to 16.30%)   - documentation 0% (IQR -1.0% to 1.3%)   - other 1% (IQR 0.8% to 8.5%)   - larger effects if best outcome is chosen - median improvement in dichotomous clinical endpoints 2.5% (1.3% to 4.2%) - general   - effect sizes larger with quasi-randomized (vs. randomized), in-patient (vs. outpatient), reminders alone (vs. part of multifaceted)   - no difference in effect size regardless of baseline compliance or specific tactics of reminder system |
| **Local Opinion Leader Interventions, n = 1** | | | | |
| Doumit (2006) [31] | Local opinion leaders: effects on professional practice and health care outcomes | RCT (12) | - improve clinical practice across clinical conditions - mixed population with cancer - participants – physicians, nurses, midwives - local opinion leaders responsible for leading educational initiatives | - decrease in rates of non-compliance:   - LOL vs. None: median ARD 7% (-6% to 12%)   - LOL vs. Single Intervention: median ARD 14% (12% to 17%)   - LOL + Additional Intervention vs. AI only: median ARD 9% (2% to 25%)   - LOL as part of multifaceted approach vs. None: median ARD 6% (1% to 14%) - general   - insufficient data to inform most effective method of choosing LOL or educational tactic   - poorer quality studies yielded bigger effect sizes than higher quality studies |
| **Tailored Interventions, n =1** | | | | |
| Baker (2010) [24] | Tailored interventions to overcome identified barriers to change: effects on professional practice and health care outcomes | RCT (26) | - improve treatment decisions across and clinical outcomes across clinical conditions - no cancer - participants - physicians, nurses, pharmacists, and health teams - tailored interventions designed to mitigate identified barriers | - pooled OR 1.54 (95% CI, 1.16 to 2.01) in favour of tailoring (binary clinical outcomes) - summary   - 8/14 studies demonstrated benefit of tailoring   - 2/14 studies demonstrated some benefit of tailoring for some outcomes   - 4 of 14 studies showed no benefit of tailoring - general   - unclear how tailoring was done in many cases (or if it was done)   - little data if tailoring overcame barriers it aimed to mitigate   - no study attributes (quality attributes, level of tailoring, complexity of behavior) were associated with interventions’ effectiveness |
| **Clinical Pathways, n =1** | | | | |
| Rotter (2010) [46] | Clinical pathways: Effects on professional practice, patient outcomes, length of stay and hospital costs | Various comparison studies (27) | - impact on professional practice, patient outcomes, length of stay, and costs - mixed population including cancer - participants - physicians, nurses, allied health providers - varied formats, modalities, adherence to defining elements | - CP alone vs. usual care   - in-hospital complications: reduced (OR 0.58; 95% CI 0.36-0.94)   - documentation: improved (OR 13.65; 95% CI 5.38 to 36.64)   - rates of hospital re-admission: n.s.   - rates of in-hospital mortality: n.s.   - length of stay: most studies reported reduction   - hospital costs/charges: WMD = $-4919 US - CP as part of multifaceted intervention vs. usual care   - no significant differences on outcomes |
| **Guidelines in Professions Allied to Medicine, n = 1** | | | | |
| Thomas (2009) [53] | Guidelines in professions allied to medicine. | Various comparison studies (18) | - impact of guidelines on processes of care, outcomes of care, and costs - mixed population including cancer - participants - nurses, allied health providers - varied formats, modalities, adherence to defining elements | - guidelines + dissemination/implementation strategies vs. no guidelines with dissemination or implementation strategy   - processes of care: improvements with at least one indicator in 3 of 5 studies with outcome   - outcomes of care: improvements with at least one indicator in 6 of 8 studies - insufficient evidence to advise on specific guideline dissemination or implementation tactics - insufficient evidence to advise on financial impact to health care system |
| **Discharge Planning from Hospital to Home, n = 1** | | | | |
| Shepperd (2010) [49] | Discharge planning from hospital to home | RCT (21) | - impact of DP on length of stay, readmissions, patient outcomes, satisfaction, costs - mixed population including cancer - participants - patients, nurses, pharmacists, physicians - varied formats, modalities, adherence to defining elements but most had assessment, implementation of plan, and monitoring - impact on mortality, health outcomes and cost are less certain | - hospital length of stay: small reduction (MD -0.91, CI 95% -1.55 to -0.27) - readmission: small reduction (RR 0.85: CI 95% 0.74 to 0.97) - satisfaction: 3 studies report increased patient satisfaction - impact on mortality, health outcomes and cost are less certain |

NB: SR = systematic review, RCT = randomized controlled trial, DS = descriptive, non-RCT = non-randomized controlled trial, RT = non-controlled randomized trial, TA = tandem assignment, LR = literature review, MM = mixed methods, 2x2 FC = 2x2 factorial comparison, NS = not specified, CRCT = cluster RCT, CCT = controlled clinical trial, TS = time series, PC = prospective cohort, RC = retrospective cohort, CS = cross-sectional, CCCT = cluster controlled clinical trial, QEL = quasi-experimental using linear modeling, CE = cost-effectiveness, RS = relationships, CBA = controlled before-after, OBS = observational, CC = case control
