# Supplementary material for: The landscape of knowledge translation interventions in cancer control: What do we know and where to next? A review of systematic reviews
Source: Implement Sci. 2011 Dec 20;6:130. doi: 10.1186/1748-5908-6-130 (PMC3284444; doi:10.1186/1748-5908-6-130)
Supplement: Additional file 5 — Interventions aimed at organizations. Table of data on each intervention aimed at organizations [file 1748-5908-6-130-S5.DOC]

Additional file 5: Interventions aimed at organizations

| **SR 1^st^ Author (Year)** | **Title of SR** | **Study designs in review (#)** | **Intervention details** | **Results** |
| --- | --- | --- | --- | --- |
| **Changing Length of Consultation Interventions, n = 1** | | | | |
| Wilson (2009) [55] | Effects of interventions aimed at changing the length of primary care physicians' consultation (Review) | RCT (2)  CRCT (2)  CCT (3) | - to improve clinical outcomes, change behaviour, improve safety, and increase satisfaction - mixed population no cancer - target – physicians - changing length of consultation between primary care provider and patient | - satisfaction   - ¼ studies showed n.s. trend in favour of longer appointment   - ¼ studies showed significant trend in favour of longer appointment   - ½ failed to detect any effects - no changes in physician behavior |
| **Routine Standard Assessment Interventions, n = 1** | | | | |
| Goldberg (2007) [36] | Pain Management in Hospitalized Cancer Patients: A Systematic Review | RCT (1)  cohort (1)  pre-post (7)  QEL (1) | - improve assessment and documentation of pain - cancer patients only - participants – physicians, nurses - routine assessment and documentation of patients’ pain symptoms | - many studies showed improved documentation of patient symptoms - many studies showed improvement in patient and staff satisfaction with pain management - some studies show improved prescribing practices - no studies showed improvements in pain scores or pain severity |
| **Chronic Care Model Interventions, n = 1** | | | | |
| Coleman (2009) [29] | Evidence on the chronic care model in the new millennium | RCT (11)  MA (14),  CE (6)  QE (30)  RS (21) | - redesign ambulatory care and evaluate practice changes - mixed population with cancer - target – physicians, nurses - various system changes to practice environment by modifying 4+ elements of chronic disease model | - 48 practice changes over 6 CCM elements reported across the studies - 34 practice changes sustained over 1 year - all RCTs found implementation of CCM resulted in at least some process improvements |
| **Models-of-Care/Integrated Care Related Interventions, n =5** | | | | |
| Lewis (2009) [42] | Follow-up of cancer in primary care versus secondary care: systematic review | RCT and non-RCT (13) | - improve patient care - cancer patients only - target – physicians - arrangement and integration of primary and specialist care | - no statistically significant differences between the intervention groups on psychological morbidity, QOL, recurrence rate, survival - primary care physicians models less expensive than specialists |
| Smith (2008) [52] | Does sharing across the primary-specialty interface improve outcomes in chronic disease? A systematic review | RCT (10)  CRCT (9) CBA (1) | - improve care of patients between health care professionals and patients - mixed population with cancer - participants - primary care physicians, specialists, nurses, primary care-secondary care, specialist team - system to continue collaborative clinical care between primary and specialty care practitioners | while outcomes favour shared care, findings not statistically significant   - recovery from depression: RR 1.49 (.09, 2.43) 95% CI - appropriate medication: RR 1.21 (1.01, 1.44) 95% CI - medication use = RR 1.29 (1.21, 1.36) 95% CI |
| Beach (2006) [25] | Improving health care quality for racial/ethnic minorities: a systematic review of the best evidence regarding provider and organization interventions | CCT (2) | - improve provision of services by way of expanding nursing roles - mixed population with cancer - target – nurse practitioners, nurses - nurses specifically trained to do diagnostic workups | - both studies showed improvements in provision of preventive services to patients |
| Goldberg (2007) [36] | Pain Management in Hospitalized Cancer Patients: A Systematic Review | Various RC, CS, OBS (10) | - to improve clinical outcomes and quality of care - cancer patients only - target – nurses - referral to nurses who are specifically trained in pain and symptom management | - all studies showed patient experience improved with specialized pain and palliative care consultation |
| Scheuner (2008) [48] | Adult Diseases: A Systematic Review Delivery of Genomic Medicine for Common Chronic Adult Diseases: A Systematic Review | RCT (4)  other (64) | - improve clinical outcomes - mixed population with cancer - participants – patients, physicians, other health care providers - formal integration of genomic services including various clinicians, nurses, policy makers, genetic counselors and professional associations | - consultation that included genetic content did not have a negative effect on patient self-efficacy, self-control or body weight |
| **Shared Care Tactic Interventions, n =1** | | | | |
| Smith (2007) [51] | Effectiveness of shared care across the interface between primary and specialty care in chronic disease management. | RCT (10)  CRCT (9)  CBA (1) | - improve care of patients between health care professionals and patients - mixed population with cancer - participants - primary care physicians, specialists, nurses, primary care-secondary care, specialist team - various shared care tactics | no statistically significant differences favouring one shared care tactics over another.   - mental health: standard effect size range 0.07 to 0.66 - psychology health: SES range -0.19 to 0.34 - hospital admission outcomes (readmission rates, mean and median number of admission, total hospital bed days, bed days per patient): SES range -0.22 to 0.72 - patient satisfaction: SES range 0 to 0.28 - increases in disease-related visits: SES range 0.03 to 0.32 - medical adherence use: SES range 0 to 0.63 |
| **Health Information Technology Interventions, n = 1** | | | | |
| Chaudhry (2006) [28] | Systematic review: impact of health information technology on quality, efficiency, and costs of medical care | RCT (25)  non-RCT TS (4)  PC (3)  CC (4)  CS (1)  CCT (4)  pre-post (10)  case series (4)  other NS (202) | - to improve clinical outcomes, change behaviour, improve safety - mixed population with cancer - target – physicians, nurses, pharmacists, clerical staff - nurses and doctors using patient EMR systems (in-patient and ambulatory) | - improvements in processes of care delivery: range 5% to 66% (most 12%-20%) - improvements in preventive health care delivery: range 12% to 33% - decreases in secondary preventive care for complications related to hospitalization: range 0.4% to 5% - improvements in clinical monitoring and large-scale screening and aggregation of data:   - increase in crude mortality from +2.45%   - increase in drug event identification from 0.04% to 2.4%   - decrease in adverse drug event rate from 7.6% to 2.2% - improvements in identification of infectious disease outbreaks: range 14% to 29% - decrease in identification time of infectious disease: from 130 hours to 46 hours - decrease in adverse events: 17% - decrease in non-intercepted serious medical errors: 86% - improvements in drug dosing: range 12% to 21% |

NB: SR = systematic review, RCT = randomized controlled trial, DS = descriptive, non-RCT = non-randomized controlled trial, RT = non-controlled randomized trial, TA = tandem assignment, LR = literature review, MM = mixed methods, 2x2 FC = 2x2 factorial comparison, NS = not specified, CRCT = cluster RCT, CCT = controlled clinical trial, TS = time series, PC = prospective cohort, RC = retrospective cohort, CS = cross-sectional, CCCT = cluster controlled clinical trial, QEL = quasi-experimental using linear modeling, CE = cost-effectiveness, RS = relationships, CBA = controlled before-after, OBS = observational, CC = case control
